# Brain space image reconstruction of functional near-infrared spectroscopy using a Bayesian adaptive fused sparse overlapping group lasso model

**DOI:** 10.1117/1.NPh.10.2.023516

**Published:** 2023-02-10

**Authors:** Xuetong Zhai, Hendrik Santosa, Robert T. Krafty, Theodore J. Huppert

**Affiliations:** a University of Pittsburgh, Department of Electrical and Computer Engineering, Pittsburgh, Pennsylvania, United States; b University of Pittsburgh, Department of Radiology, Pittsburgh, Pennsylvania, United States; c Emory University, Department of Biostatistics and Bioinformatics, Atlanta, Georgia, United States; d University of Pittsburgh, Clinical Science Translational Institute, and Center for the Neural Basis of Cognition, Department of Electrical and Computer Engineering, Department of Bioengineering, Pittsburgh, Pennsylvania, United States

**Keywords:** functional near-infrared spectroscopy, image reconstruction, adaptive fused sparse overlapping group lasso, Bayesian hierarchical modeling

## Abstract

**Significance:**

Functional near-infrared spectroscopy (fNIRS) is a noninvasive technology that uses low levels of nonionizing light in the range of red and near-infrared to record changes in the optical absorption and scattering of the underlying tissue that can be used to infer blood flow and oxygen changes during brain activity. The challenges and difficulties of reconstructing spatial images of hemoglobin changes from fNIRS data are mainly caused by the illposed nature of the optical inverse model.

**Aim:**

We describe a Bayesian approach combining several lasso-based regularizations to apply anatomy-prior information to solving the inverse model.

**Approach:**

We built a Bayesian hierarchical model to solve the Bayesian adaptive fused sparse overlapping group lasso (Ba-FSOGL) model. The method is evaluated and validated using simulation and experimental datasets.

**Results:**

We apply this approach to the simulation and experimental datasets to reconstruct a known brain activity. The reconstructed images and statistical plots are shown.

**Conclusion:**

We discuss the adaptation of this method to fNIRS data and demonstrate that this approach provides accurate image reconstruction with a low false-positive rate, through numerical simulations and application to experimental data collected during motor and sensory tasks.

## 1 Introduction

Functional near-infrared spectroscopy (fNIRS) is a noninvasive brain imaging technique, which uses scalp-placed optical sensors to record changes in the optical absorption of the underlying tissue and to infer changes in blood flow and oxygenation in the brain during cognitive tasks.^1^ A limited spatial localization of these changes can be made by image reconstruction using the discrete set of measurements made between optical sources and detectors. However, this is a greatly under-determined problem with typically hundreds of unknown parameters in the brain (image) space compared to the dozens of actual measurements. This problem is also illposed; having multiple solutions of the underlying image that would generate indistinguishable channel-space measurements. Thus, the reconstruction of fNIRS data into brain-space images requires additional constraints through mathematical regularization and/or additional prior information.

Studies on solving the optical inverse model in recent years have brought continuous improvements.^2^ Most of the developments involved restricted maximum likelihood (ReML),^3^ maximum entropy on the mean (MEM),^4^^–^^6^ and depth compensation^7^ to weighted minimum norm (WMN) or Tikhonov regularization. According to our experience, these methods tend to overestimate the false positive rate due to the nature of the regularization approaches.^8^ Previous studies^9^^–^^11^ built Bayesian models incorporating prior information of cortical/scalp areas, sensitivity normalization, and so on, for removing scalp artifact, improving depth accuracy and spatial resolution, and multisubject and multitask experiments. However, the prior spatial information of the brain anatomy of cerebral functional areas has never been properly used in current fNIRS image reconstruction methods.

In this work, we describe an adaptive fused sparse overlapping group lasso (a-FSOGL) regularization approach for fNIRS image reconstruction. The a-FSOGL model uses brain-space voxel grouping priors, for example from atlas-based regions-of-interest, to regularize the image reconstruction process. To make a better use of the prior information, we develop a Bayesian framework to solve this model by incorporating the prior information with appropriate statistical distributions. The framework is built based on previous studies^12^^–^^16^ of the Bayesian lasso model and its extensions. Our model extends the Bayesian lasso models a step further by combining existing models and involving more prior parameters. In this paper, we will first briefly review the principles of the optical forward and inverse models, then derive the Bayesian model of a-FSOGL (Ba-FSOGL) and its associated statistical properties before demonstrating the approach using simulated fNIRS measurements and experimental data.

The paper is organized as follows. An overview of the optical forward model is provided in the theory section (Sec. 2). In the methods part (Secs. 3 and 4), we then describe the Ba-FSOGL model, the simulation configurations, and the experimental data collection. The results of the image reconstruction and statistical inference are shown in Sec. 5, and we finally discuss the findings from the results and the limitations of the model in Sec. 6. In the simulation study, we focus on the example of a nearest-neighbor bilateral fNIRS probe over the forehead and examine the ability to infer changes in frontal and dorsolateral brain regions as defined by atlas-based Brodmann area (BA) parcellations, however, the experimental study demonstrates that this approach is applicable to any brain space parcellation model as prior information.

## 2 Theory

### 2.1 Optical Forward Model

The optical forward model has been described in detail in previous literature.^1^ Here we only discuss it briefly. In an experiment using fNIRS, a set of light sources and detectors is placed on the scalp surface. The light is emitted from each source and transmitted through the tissue at two or more wavelengths. The light spreads after it is sent into the brain due to the scattering property of the tissue. The propagation path of light through brain tissue depends on its anatomical structure, including scalp, skull, cerebral spinal fluid (CSF), gray/white matter, and so on, which can be approximated by a diffusion-based random walk of the photons of light and modeled through Monte Carlo, finite difference, finite element, or boundary element methods. During brain activity, the fluctuation of the blood flow in the cerebral cortex leads to the alteration of the hemoglobin concentration and consequently changes the light absorption ability of the brain tissue. The optical forward model describes the relationship between the optical density changes recorded by light source-detector pairs on the surface and the hemoglobin concentration changes in the underlying tissue. For a typical amount of hemoglobin concentration change, the change in the optical density at a given wavelength l can be modeled by the modified Beer–Lambert law as

$$\Delta OD_{i,j}^{l}=X_{i,j}^{l}[\varepsilon_{\mathrm{HbO}}^{l}(\Delta [\mathrm{HbO}]+\xi_{\mathrm{HbO}})+\varepsilon_{\mathrm{HbR}}^{l}(\Delta [\mathrm{HbR}]+\xi_{\mathrm{HbR}})]+\nu_{i,j}^{l},$$ (1)

where $X_{i,j}$ is the Jacobian of the optical measurement model describes the total absorption by each voxel along the traveling path of light transmitted between the source to the detector pair (i,j). $\varepsilon_{\mathrm{HbX}}$ is the molar extinction coefficient, Δ[HbX] is the vector containing the hemoglobin changes, and $\xi_{\mathrm{HbX}}$ is the physiological noise vector, in which HbX represents HbO or HbR for oxy- and deoxy-hemoglobin, respectively. $\nu_{i,j}$ is the additive measurement space noise. Note that $X_{i,j}$, Δ[HbX], and $\xi_{\mathrm{HbX}}$ are vectors with a length that is the same as the number of voxels. For measurements between multiple channels (source-detector pair) at multiple wavelengths, the model can be written in a compact linear expression

y=X(β+ξ)+ν (2)

where y contains the measurements between all source-detector pairs and β includes oxy- and deoxy-hemoglobin concentration changes at each voxel in the brain image.

$$y=[\begin{array}{c} \Delta OD_{i,j}^{l_{1}}\\ \Delta OD_{i,j}^{l_{2}}\\ \vdots \end{array}]\;\text{and}\;\beta =[\begin{array}{c} \Delta [\mathrm{HbO}]\\ \Delta [\mathrm{HbR}] \end{array}].$$ (3)

Thus, y and ν are the measurement and measurement-space noise vector, respectively, having a length of N, which equals to the number of source-detector pairs times the number of wavelengths. β and ξ are two vectors containing the parameters of interest – the hemoglobin concentration changes – and the physiological noise at each voxel, respectively. Both of the vectors have a length of P, which equals to the double of the total number of voxels (HbO and HbR for each voxel). X is a N×P matrix whose each row contains the Jacobian for a channel.

### 2.2 Inverse Problem of fNIRS Image Reconstruction

The fNIRS brain image is obtained by solving Eq. (2), which is a highly dimensional underdetermined (P≫N) inverse problem since we usually have hemoglobin changes at thousands of voxels to estimate but only tens of measurements available, i.e., the number of unknowns is extremely greater than that of the knowns. Regularization approaches are commonly used for stabilizing the solution of the inverse problem by minimizing an objective function including an additional penalty term to the least-squares cost function, which can be represented as

β^=arg minβ‖y−Xβ‖Cν−12+λJ(β), (4)

where λ≥0 is a tuning parameter adjusting the weight of the regularization. ${\left\|y-X\beta \right\|}_{C_{\nu }^{-1}}^{2}$ is the least-squares cost function, in which $C_{\nu }$ is the covariance matrix of the channel space error ν and ${\left\|A\right\|}_{B}^{2}=A^{T}\mathrm{BA}$ denotes the weighted $\ell_{2}$ norm calculation. J(β) is the penalty term applying the constraints on the sparsity and/or structure to the estimation of β, which allows to incorporate prior information about the elements in β. Some commonly used penalties terms are shown in Table 1.

**Table 1:** Summary of commonly used penalties terms for regularization approaches and their properties.

Penalty	J(β)	Property
Lasso^17^	${\left\|\beta \right\|}_{1}$ where ${\left\|\beta \right\|}_{1}=\overset{P}{\underset{p=1}{\sum }}|\beta_{p}|$ denoting the $\ell_{1}$ norm $\beta_{p}\in \beta$	Shrink some parameters to exact 0; proper for sparse solution space; no analytical solution
Tikhonov^18^	${\left\|\beta \right\|}_{C_{\beta }^{-1}}^{2}$ where $C_{\beta }$ is the covariance matrix of β coefficients	Cannot shrink parameters to exact 0; have a unique analytical solution for a specific tuning parameter; easy to interpose covariance of β
Elastic net^19^	$\gamma {\left\|\beta \right\|}_{1}+(1-\gamma ){\left\|\beta \right\|}_{C_{\beta }^{-1}}^{2}$ where γ∈[0,1]	A weighted combination of lasso and Tikhonov regularization
Fused lasso^20^	$\gamma {\left\|\beta \right\|}_{1}+(1-\gamma ){\left\|D\beta \right\|}_{1}$ where D encodes the spatial structure	Shrink the difference between neighboring elements in β to 0, i.e., constraining them to be equal, in addition to the lasso penalty
Group lasso^21^	$\overset{G}{\underset{g=1}{\sum }}\sqrt{p_{g}}{\left\|\beta_{g}\right\|}_{C_{\beta_{g}}^{-1}}$ where β is split into G groups, $\beta_{g}$ contains the elements in the g’th group, and $C_{\beta_{g}}$ is the covariance matrix of $\beta_{g}$	The penalty is intermediate between lasso and Tikhonov; perform variable selection at the group level
Sparse group lasso^22^	$\gamma {\left\|\beta \right\|}_{1}+(1-\gamma )\overset{G}{\underset{g=1}{\sum }}\sqrt{p_{g}}{\left\|\beta \right\|}_{gC_{\beta_{g}}^{-1}}$	A weighted combination of lasso and group lasso; perform variable selection at both individual and group level

### 2.3 Prior Information on Cerebral Anatomy and Hemodynamics

In an evoked-task study, the observable brain activity usually only appears within a certain area. The location of the active region depends on the type of the task, e.g., Broca’s area is evoked in most speech- or language-related tasks,^23^^–^^25^ and voluntary movement- or control-involved tasks often activate the motor cortex area.^26^^,^^27^ Thus, for a specific task, one can have the prior information on the potential areas of interest and the anatomical divisions, e.g., the movement of different parts of the body can be mapped to the motor cortex according to the motor homunculus.^28^^,^^29^

Brain activity leads to a growth in blood flow and oxygen consumption. The growth in blood flow increases the blood volume, brings more HbO, and moves more HbR away, and the growth in oxygen consumption results in an increase in the concentration of HbR and a decrease in that of HbO. The two effects jointly increase the concentration of HbO and decrease that of HbR during the brain activity. It is also known that the change in the HbR concentration is smaller than that in the HbO concentration.

## 3 Methods

In this paper, we apply an adaptive fused sparse overlapping group lasso (a-FSOGL) regularization to the inverse problem of fNIRS image reconstruction and validate the model via numerical simulations. This section describes the model and the Bayesian algorithm to solve the model in detail followed by the procedures of the simulation and evaluation.

### 3.1 Adaptive Fused Sparse Overlapping Group Lasso

The a-FSOGL is an extension of the combination of fused and sparse group lasso, which can handle overlapping groups of β and allows different tuning parameters for each group. As shown in Table 1, the sparse group penalty can perform variable selection at both individual and group level. Thus, this penalty term incorporates the prior information on the potential areas of interest and the anatomical divisions by splitting β into groups, which includes/excludes each area entirely and allows some individual voxels to be excluded/included. The elements of a group of β correspond to the HbO and HbR concentration changes at the voxels in a division of the potential area. The covariance matrix of β can be used to apply the hemodynamics prior to constraining the HbO and HbR concentration changes at the same voxel to be anticorrelated. In addition, since the hemoglobin concentration changes within a group are not independent, the fused lasso penalty term is added to minimize the hemoglobin concentration changes at neighboring voxels. A previous study^30^ showed that the variable selection exhibited by the lasso model is inconsistent except for a specific nontrivial condition and develops the adaptive lasso model to reach consistent variable selection by using different tuning parameters for each coefficient. For the same reason, adaptive fused lasso^31^ and adaptive groups^32^ have also been proposed. Similarly, here we also use the adaptive version of regularization.

**Fig. 1:**
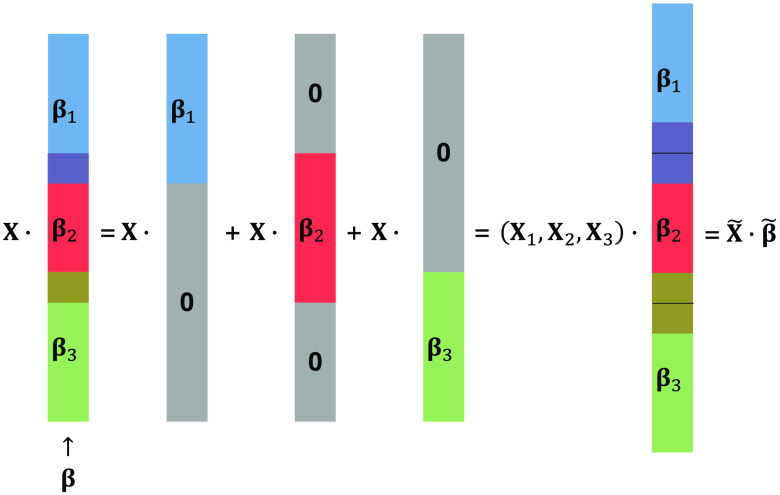
An example demonstrating the equivalence between an overlapping group lasso and a regular group lasso with duplicated covariates. $\beta_{1}$ (blue), $\beta_{2}$ (red), and $\beta_{3}$ (green) are the three groups of β where there exist overlaps between $\beta_{1}$, $\beta_{2}$ (purple) and $\beta_{2}$, $\beta_{3}$ (yellow). $X_{1}$, $X_{2}$, and $X_{3}$ are the submatrix of X corresponding to $\beta_{1}$, $\beta_{2}$, and $\beta_{3}$, respectively. X and β are constructed by concatenating $X_{1}$, $X_{2}$, $X_{3}$ and $\beta_{1}$, $\beta_{2}$, $\beta_{3}$ with duplicating the overlapping parts, respectively.

It is difficult to precisely split the cortex into regions of interest (ROI) at the voxel level since some voxels can potentially belong to multiple groups depending on how the atlas is defined. For example, we found the specific parcellation of BAs which came from the Talairach Daemon atlas,^33^ which is used in the simulation study of this paper, assigns some voxels into multiple groups, especially those around and on the border between two areas, i.e., the neighboring two groups overlap to each other. Previous studies^34^^,^^35^ demonstrated that the overlapping group lasso is equivalent to a regular group lasso by duplicating the covariates belonging to multiple groups as shown in Fig. 1.

From previous studies,^36^^,^^37^ we can obtain the covariance matrix of the measurements error, $C_{\nu }$, from the channel space analysis of the given fNIRS dataset. To reduce the number of optimization parameters in the model, the correlation of the error term can be removed through whitening transformation. Let W denote the Cholesky decomposition of $C_{\nu }^{-1}$, i.e., $W^{T}W=C_{\nu }^{-1}$. X and y can be transformed via $X^{*}=\mathrm{WX}$ and $y^{*}=\mathrm{Wy}$. The optimization problem using the transformed variables is equivalent to the original one involving the covariance matrix. To maintain conciseness of the notation, X, y, and β will represent the expanded and decorrelated variables, ${\overset{˜}{X}}^{*}$, $y^{*}$, and $\overset{˜}{\beta }$ in the remaining part of this paper.

The a-FSOGL is proposed to estimate β by minimizing the cost function shown as

β^=arg minβ{‖y−Xβ‖2+∑g=1Gλg[θγ‖βg‖1+(1−γ)‖Dgβg‖1+(1−θ)γ‖βg‖Cβg−1]}. (5)

**Fig. 2:**
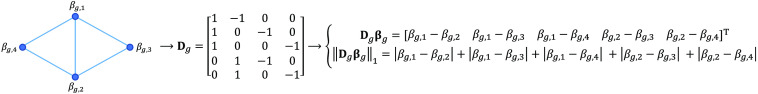
A simple example of $D_{g}$. The left diagram shows the structure of $\beta_{g}$ where there are four elements (represented by the solid circles) and five connected pairs (connections represented by the solid lines). Thus, $D_{g}$ is a 5×4 matrix, in which each row represents a connected pair by assigning 1 and −1 to the columns corresponding to the indices of the two elements of the pair and 0 to the remaining columns. Finally, ${\left\|D_{g}\beta_{g}\right\|}_{1}$ provides the summation of the absolute differences between βs of each paired connection in the spatial structure.

Here $\lambda_{g}\geq 0$ is the tuning parameter for the g’th group controlling the overall level of regularization, and θ,γ∈[0,1] are the two parameters jointly define the weights of the three penalty terms.^37^ When θ or γ=0 or 1, some penalty terms are dropped and the minimization degenerates into a subset of a-FSOGL. For example, when θ=1 and γ=1, the model reduces to a standard adaptive lasso, and so on. Let $m_{g}$ denote the number of elemets in $\beta_{g}$ and $q_{g}$ denote the number of connected voxel pairs in $\beta_{g}$. Note that $m_{g}$ equals double of the number of voxels (HbO and HbR for each voxel) in the group, and $\sum_{g=1}^{G}m_{g}=P$. Then $D_{g}$ is a $q_{g}\times m_{g}$ matrix encoding the spatial structure of $\beta_{g}$. A simple example of $D_{g}$ is shown in Fig. 2.

Note that in this paper we arrange the HbO changes as the first $\frac{m_{g}}{2}$ rows of $\beta_{g}$ and the HbR changes as the second half rows. $D_{g}$ can be decomposed into four $\frac{q_{g}}{2}\times \frac{m_{g}}{2}$ submatrices. The two submatrices on the diagonal are identical, and each of them represents the spatial structure of the voxels. The two off-diagonal submatrices are both zero matrix as HbO and HbR changes are not expected to be equal.

The number of parameters needs to be optimized in a-FSOGL is usually >1000 including β and its covariance matrix. Searching in such a high-dimensional solution space and maintaining the semipositive definiteness of the covariance matrix are challenging using the conventional gradient-based minimization algorithms. Alternatively, penalized least squares estimators of the form of Eq. (4) have an alternative interpretation as the Bayes posterior mode under a suitably selected hierarchical model. Thus, we estimate via Bayesian hierarchical modeling, which is detailed in the Supplemental Material.

### 3.2 Statistical Inference

In a frequentist framework, statistical inference of lasso-based model is usually unnecessary since insignificant variables are forced to be zero. However, the probability of exactly hitting any specific number from a continuous distribution is zero. The samples from the Gibbs sampler cannot give exact zero estimates no matter how small they are. Statistical inference is required to determine the significance of variables in the Bayesian framework.

Two interval-based approaches^14^ are used for the inference on every individual variable, $\beta_{p}$, in this study. First, $\beta_{p}$ is statistically significant if its credible interval (CI) excludes 0 and insignificant otherwise. Second, we calculate the posterior probability that $\beta_{p}$ is within the scaled neighborhood interval $\left[-\sqrt{\mathrm{var}(\beta_{p}|X,y)},\sqrt{\mathrm{var}(\beta_{p}|X,y)}\right]$. $\beta_{p}$ is considered to be insignificant if this probability exceeds a certain threshold and significant otherwise. In addition to the inference on individual variables, we also perform statistical inference on the significance of the variables in a group, $\beta_{g}$, as an entirety. The CIs of the random variable $\beta_{g}^{T}\Sigma_{g}^{-1}\beta_{g}$ for all groups are compared. If the two intervals overlap with each other, the two groups are not significantly different, and vice versa.

Let α denote the level of the CI and η denote the probability threshold described above. The selection of α and η affects the statistical inference. Previous studies show 95% (α=0.05) CIs are usually too wide. Setting large values for α and η—narrow CI and difficult threshold—would lead to high sensitivity but low specificity, and vice versa. The previous study^14^ suggests moderate values α=0.5 and η=0.5 in practice, which are used in this paper.

### 3.3 Simulation Study

In this paper, we validate the proposed model by applying Ba-FSOGL to simulated fNIRS datasets and comparing the reconstructed images with the simulated truth images. The fNIRS datasets are simulated using the Brain AnalyzIR toolbox.^38^ In each iteration of simulation, brain activities are simulated within a specific BA. The BA membership of each voxel of the atlas is used as the anatomical prior information for the image reconstruction.

#### 3.3.1 Probe configuration

**Fig. 3:**
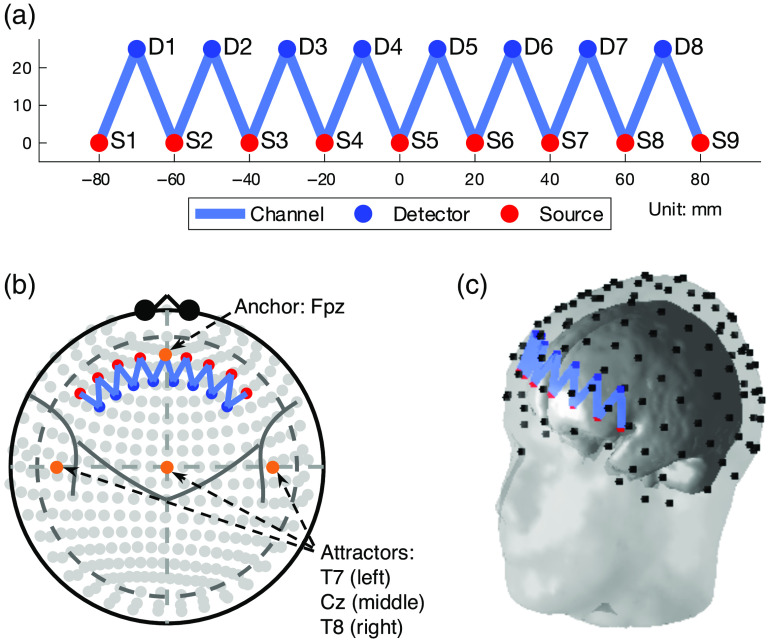
The topology of the probe used in the simulation: (a) The 2D layout in the Cartesian coordinate system, (b) the registered probe with 10-20 system, and (c) the 3D geometry of the probe registered on an example head.

The probe used in the simulation study is the same as the one used in a previous publication. It contains nine light sources and eight detectors. Sources and detectors are respectively aligned, and the distances between neighboring sources-detectors are 20 mm. The source alignment is placed 25 mm apart from the detector alignment. The optical density is only measured between the nearest source-detector pairs. Hence, there are 32 (two wavelengths, 16 for HbO and the other 16 for HbR) channels defined in this probe. Figure 3(a) shows the 2D layout of the probe in the Cartesian coordinate system. The registration of the probe is constrained by an anchor and three attractors. Similar to the use of these terms in the AtlasViewer program,^39^ in the Brain AnalyzIR toolbox,^38^ an anchor forcibly places a point of the probe layout [Fig. 3(a)] on the 10-20 system, and an attractor defines the direction to pull the probe. In this case, the origin of the probe (0, 0) in the 2D layout is anchored to the site Fpz. Three attractors are placed at positions (±200,0) and (0, 100) in the 2D layout and attached to T7, T8, and Cz, respectively, which define three forces pulling the probe along negative/positive horizontal axis and positive vertical axis to T7, T8, and Cz. An iterative least-squares minimization algorithm is used to register the probe based on the optimal source-detector pair spacings and the location of the anchor/attractor. Unit vectors are constructed using attractors to provide direction, which is updated with every iteration of the algorithm. The registered probe is shown in Figs. 3(b) and 3(c) using 10-20 (Mercator) projection and 3D geometry on an example head.

#### 3.3.2 Preselection on regions-of-interest

The probe used in this study has a low-density style configuration that is frequently used in fNIRS studies due to the ease and economicalness of use. This style of probe has “blind-spots” because of regions of low sensitivity to underlying brain activity.^40^ The brain activity within the areas falling into blind spots cannot be detected by the probe. Thus, we need to determine the detectable regions of interest before the simulation study.

**Fig. 4:**
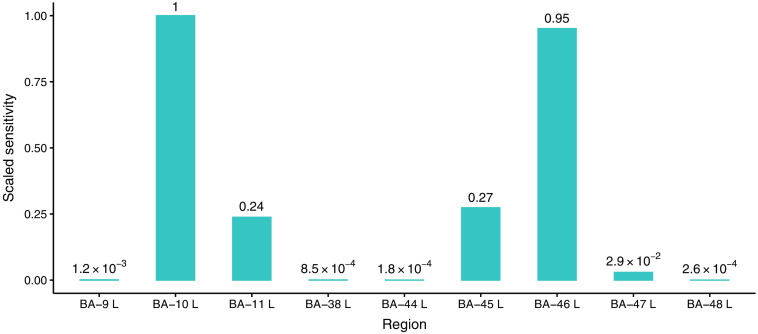
Scaled sensitivity of each BA to the probe. The values in the plot are calculated by summing up the forward model of all voxels within each area, then scaling the values by the largest sensitivity among all areas. Due to the symmetry of the brain, only the left regions are shown here. Note that (1) the scaled sensitivities in this plot are calculated based on the specific probe in this study; (2) voxels apart from the nearest channel further than 5 cm are excluded, so the entirely excluded regions are not shown in this plot (e.g., BA-39.).

Figure 4 is a bar chart for the relative sensitivity to each BA using the probe. Due to the symmetry of the probe and the two brain hemispheres, we only simulate activities within the BAs on the left hemisphere. Thus, the BAs on the right side are omitted in Fig. 4. The values in the plot are calculated by summing up the forward model of all voxels within each area, then scaling the values by the largest sensitivity among all areas. From the figure, we can see that the probe is most sensitive to BA-10 followed by BA-46, BA-45, and BA-11. For the remaining regions, considering the sensitivities are less than $\frac{1}{30}$ of BA-10, which means the brain activity in any one of these regions cannot survive from the physiological noise in BA-10 unless the signal-to-noise (SNR) is impractically greater than 900, a reasonable brain activity in these regions is not observable using this probe, so we will not generate brain activity in these regions. BA-11 is located at the bottom of the frontal lobe of brain, i.e., right beneath BA-10. The two regions are covered by the same source-detector pairs of the probe used in this study, and the light sent from those sources goes through both regions. A brain activity in BA-11 consequently always results in a smaller false positive (FP) in BA-10 since it is closer to the probe and regularization-based approaches tend to select variables with smaller values. Therefore, BA-11 is another region that will not be used in the simulation.

**Fig. 5:**
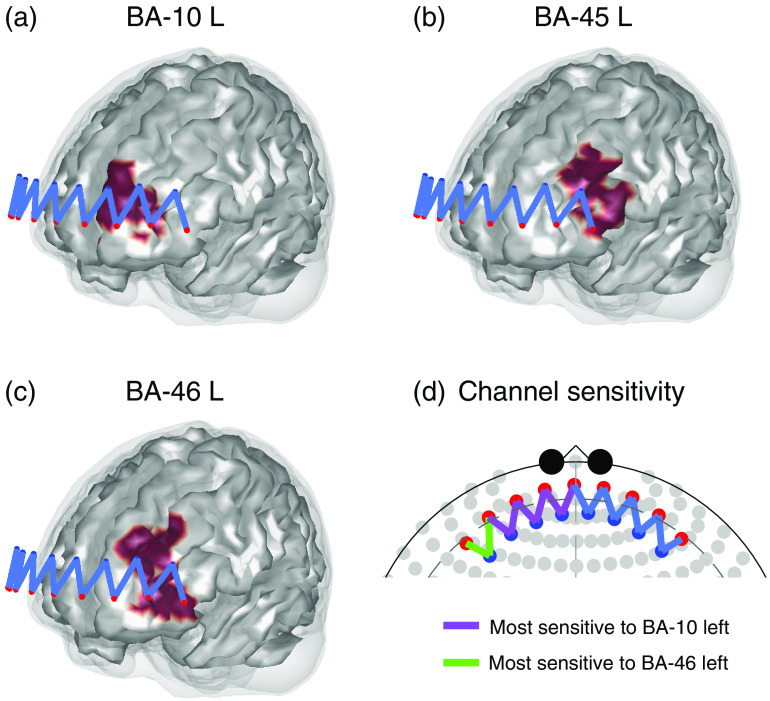
(a)–(c) The locations of left BA-10, BA-45, and BA-46 on the cortex as well as their relative positions to the probe. (d) The most sensitive area from each channel. Note that the right side is omitted due to the symmetry.

Brain activities in BA-10, BA-45, and BA-46, both left and right side, will be considered as the regions-of-interest using the probe. Figures 5(a)–5(c) show the locations of the three left regions on the cortex as well as their relative positions to the probe. Figure 5(d) demonstrates the most sensitive area from each channel where we can see the middle four channels are more sensitive to BA-10 while the lateral two channels are more sensitive to BA-46. There is no channel most sensitive to BA-45 because it is further from all channels of the probe than BA-10 and BA-46.

#### 3.3.3 Stimulus generation

The fNIRS data is simulated by adding stimulation on autoregressive noise. The time difference between two neighboring stimuli is exponentially distributed. The hemodynamic response to the stimulus is simulated using canonical hemodynamic response function. The peaks of HbO and HbR concentration changes are 7 and −2  μM (micromolar, a.k.a., μmol/L), respectively. In brief, simulated “brain” activity within the ROI (true positive) is computed and projected to fNIRS channel/measurement space via the optical forward model. The details are described in Ref. 38. In each iteration of simulations, we simulate the stimulus in only one ROIs, and both stimuli added data and the corresponding noise data will be reconstructed using Ba-FSOGL. Since the left and right three ROIs are mirrored correspondingly, only the left three regions are used to generate stimulus to avoid complexity. For each of the three regions, BA-10 left, BA-45 left, and BA-46 left, we simulate 100 datasets by adding stimulus in the corresponding regions to noise data using Brain AnalyzIR toolbox, and the 100 noise-only datasets are also retained for estimating false positive rate (FPR). To sum up, 600 datasets—300 activity-present and 300 noise-only—are simulated in this study.

#### 3.3.4 Image reconstruction evaluation

We will evaluate the reconstructed images using conventional indicators and receiver operating characteristics (ROC) performance. The two conventional indicators are mean squared error (MSE) and contrast-to-noise (CNR) defined as follows:

MSEHbX=1P/2‖βHbX−β^HbX‖22, (6)

CNRHbX=10×log10(‖β^HbX2‖22‖βHbX−β^HbX2‖22), (7)

where $\beta_{\mathrm{HbX}}$ and β^HbX are the ground truth and estimates for HbO/HbR changes from a given dataset. Note that the averaging factor of MSE is P/2 because $\beta_{\mathrm{HbO}}$ and $\beta_{\mathrm{HbR}}$ are the two halves of β with an equal length. MSE measures the average of the difference between the truth and the reconstructed images and CNR shows the ability to distinguish brain activities from the background noise.

The ROC used in this study is called ROI-ROC.^41^ Note that the term “ROI” used in this paragraph has a different definition from that in the remaining sections of this paper. Here the ROI refers to any area with a rating. In the evaluation of the image reconstruction results, two levels of ROI are used—voxel and BA level. The ROC performance of the model is evaluated per the active region. For brain activity in each of the three BAs, the estimated HbO and HbR changes at each voxel of the 200 datasets (100 activity-present and 100 noise-only) are respectively concatenated, in which the hemoglobin changes for the voxels in an active region will be considered as true positives (TP) and FPs otherwise. The values of $\beta_{g}^{T}\Sigma_{g}^{-1}\beta_{g}$ for the six BAs are concatenated with the same definitions of TP and FP from the 200 datasets. Thus, we can draw three ROC curves—two at the voxel level (HbO and HbR) and one at the BA level, in which the estimated HbO change, the negative estimated HbR change (as the HbR change in an active region is negative), and $\beta_{g}^{T}\Sigma_{g}^{-1}\beta_{g}$ are, respectively, used as the ROI–ROC rating to construct the ROI–ROC curve.

#### 3.3.5 Choosing hyperparameters and initial values

The Bayesian approach requires a reasonable selection of the hyperparameters and initial values, especially for high-dimensional problems. We will discuss how to determine these values in this section.

**Fig. 6:**
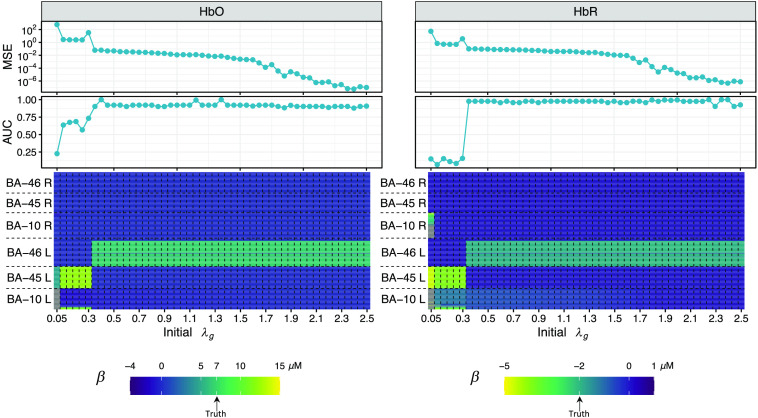
An example of image reconstruction for a simulation dataset containing brain activity within BA-46 left area. The left panel of the figure shows the image reconstruction on HbO and the right panel is for HbR. The bottom two heatmaps conclude the image reconstruction results using 50 initial $\lambda_{g}$ values from 0.05 to 2.5. Each column represents a reconstructed image using the initial $\lambda_{g}$ indicated on the horizontal axis. The image is split into six parts along the vertical axis whose ROI membership is indicated on the axis. The color of the heatmap represents the value of the HbO/HbR change. The truth values are annotated on the legends. The two line plots show the ROC AUC and MSE.

First, the hyperparameters r and s for the hyperprior distribution of $\sigma^{2}$, given by Eq. (S12) in the Supplemental Material, are determined by preliminary trials. In this subsection, we find that the magnitude of the samples of $\sigma^{2}$ should be around 0.005 so that the samples of β can fluctuate from zero but not be too large to break the Gibbs sampler. To limit $\sigma^{2}$ within a reasonable range, we set r=2500 and s=0. Then it is found that the initial value of the tuning parameter $\lambda_{g}$ can affect the image reconstruction result, although the algorithm optimizes it during the Gibbs sampling process, which is a common problem that different start points may lead an optimization process to different local optima. In this subsection, we perform channel-space ROI analysis for all ROIs before the image reconstruction following the method described in a previous study.^42^ The channel-space analysis can provide the prior information on which ROI has the most significant activity by comparing their channel-space ROI statistics. Then we apply Ba-FSOGL to the dataset to reconstruct images with multiple initial $\lambda_{g}$. Note that $\lambda_{g}$ starts from the same value for all ROIs at each time of image reconstruction. After obtaining the reconstructed images using multiple initial values, we can determine which is the best estimation based on the channel-space analysis. If no significant activity is found from any ROI (no p-value<0.05), this dataset will be considered as a noise-only dataset, for which we know the ground truth is all zeros. The initial $\lambda_{g}$ generating the minimum MSE will be selected as the final result of the image reconstruction. If significant activities are found in at least one ROI, the most significant (with the smallest p-value) ROI will be considered to contain the brain activity. Although the values of HbO and HbR changes are unknown, we can construct an ROC curve for the reconstructed image using each initial $\lambda_{g}$. In addition, the MSE for the remaining ROIs can be calculated since we know there is no activity in these ROIs and the HbO and HbR changes are expected to be zero. The optimal initial value of $\lambda_{g}$ can be selected based on the area under the ROC curve (AUC) and the MSE. Figure 6 is an example of image reconstruction for a simulation dataset containing brain activity within BA-46 left area. The channel-space analysis demonstrates that BA-46 left area is the most active one among the six Brodmann ROIs. The left panel of the figure shows the image reconstruction on HbO while the right panel is for HbR. The bottom two heatmaps conclude the image reconstruction results using 50 initial $\lambda_{g}$ values from 0.05 to 2.5. Each column represents a reconstructed image using the initial $\lambda_{g}$ indicated on the horizontal axis. The image is split into six parts along the vertical axis whose ROI membership is indicated on the axis. The color of the heatmap represents the value of the HbO/HbR change. The truth values are annotated on the legends. The four-line plots show the ROC AUC and MSE described above. From this figure, we can see that image reconstructions with initial $\lambda_{g}<0.3$ are completely off the target where a brain activity stronger (brighter color) than the simulated ground truth is estimated at a different ROI (BA-45 left instead of BA-46 left), so it is not surprising that the ROC AUCs are lower and the MSEs are higher in this range of initial $\lambda_{g}$. It is widely known that the solution for an underdetermined inverse problem is not unique. As the level of regularization increases, the optimization tends to select variables with smaller coefficients. This nature of regularization methods can be seen from this figure. Since BA-45 left is further from the probe than BA-46 left, a same measurement vector y can be obtained with a larger brain activity in BA-45 left or a smaller one in BA-46 left with different noise. Thus, the larger activity in BA-45 left is preferred by small initial $\lambda_{g}$ while the smaller on in BA-46 left is preferred by larger initial $\lambda_{g}$. To select the best initial $\lambda_{g}$, we can compare their AUCs and MSEs. As we can see from the line plots of Fig. 6, the AUCs are stable around a high level for initial $\lambda_{g}>0.5$ while the MSE continues decreasing until 2.4. Thus, the optimal initial value of $\lambda_{g}$ for this dataset is about 2.4.

A question may be raised about the search range of the initial $\lambda_{g}$. From this study, we find that the results for initial $\lambda_{g}>2.5$ are stable and similar until it is over-regularized around initial $\lambda_{g}=10$ and gives an all-zero estimation. Thus, we will omit the results for initial $\lambda_{g}>2.5$ and only select initial $\lambda_{g}$ from the range shown in Fig. 6.

Two more hyperparameters that need to be determined are θ and γ controlling the weights of the three penalty terms. These two hyperparameters can be selected based on prior knowledge and preliminary trials. For example, simulation datasets are used in this study, in which the brain activities are uniform within the active region and anticorrelation between HbO and HbR changes are properly simulated. Thus, we need a large weight for the fused and group lasso penalty terms but a small weight for the sparse penalty term. After some preliminary trials, we select θ=0.125 and γ=0.4, which assigns 0.05, 0.6, and 0.35 as the weight of the sparse, fused, and group lasso penalty term, respectively. This combination of weights results in fairly uniform brain activity and anticorrelated HbO and HbR changes. If there is little prior information on the penalty weights is known, we can still use the approach described in this section for selecting $\lambda_{g}$ to determine θ and γ.

### 3.4 Implementation of fNIRS Data Simulation and Gibbs Sampler

The simulation of fNIRS brain image data has already been implemented in the Brain AnalyzIR toolbox—an open-source MATLAB-based analysis toolbox for fNIRS data. This section describes the main components of fNIRS data simulation in the toolbox as well as the Gibbs sampler implementation.

#### 3.4.1 Forward model

The AnalyzIR toolbox provides accesses to third-party optical forward model solvers including NIRFAST,^43^^,^^44^ Mesh-based Monte Carlo (MMC^45^^,^^46^) and Monte Carlo Extreme (MCX^47^^,^^48^), which allow construction and import of individual head models from anatomical MRI volumes. We can use these solvers to generate the optical forward model with either atlas-based or individual MRI head models. However, since the computation of optical forward models is usually time-consuming and furthermore the individual-level anatomical modeling is not always available for all subjects (e.g., pediatric fNIRS studies), the default options in the AnalyzIR toolbox, which is also used in this study, utilize a presegmented head model derived from the Colin-27 atlas.^49^

#### 3.4.2 Brodmann area parcellation

The fNIRS AnalyzIR toolbox contains atlas-based parcellations of the Colin-27 atlas brain^49^ based on several packages including the automatic-anatomical labeling model (AAL2),^50^ the Freesurfer Desikan–Killiany atlas,^51^ Human–Connectome Project MSM atlas,^52^ and Broadmann area labels from both the Talairach Daemon^33^ and the MRIcron provided atlas.^53^ In this work, the Talairach Daemon labeling of the BAs was used.

#### 3.4.3 Gibbs sampler implementation

For each of the 600 datasets, we apply the proposed Ba-FSOGL model with 50 different initial $\lambda_{g}$ for image reconstruction and select the optimal estimated images following the method described in Sec. 3.3.5. The Gibbs sampler for the Ba-FSOGL model is implemented in MATLAB using its built-in random number generators for sampling from multivariate normal, inverse gamma, and inverse Gaussian distributions. For a specific fNIRS dataset with a given value of initial $\lambda_{g}$, the Gibbs sampler runs 100,000 sampling iterations, in which the first 10,000 iterations are abandoned as the burn-in period and the samples are extracted every nine iterations in the remaining 90,000 iterations to maintain the independency among the output samples as nearby samples in a Markov chain are not independent. Finally, 10,000 samples are finally retained from the Gibbs sampling process for estimating β.

## 4 Experimental Validation

In this section, we designed an experiment as a preliminary validation of our methods. The experiment included two cognitive tasks, and the data was collected from seven subjects using high-density probes. The experiment configurations are summarized in this section.

### 4.1 Instruments and Probe Configuration

**Fig. 7:**
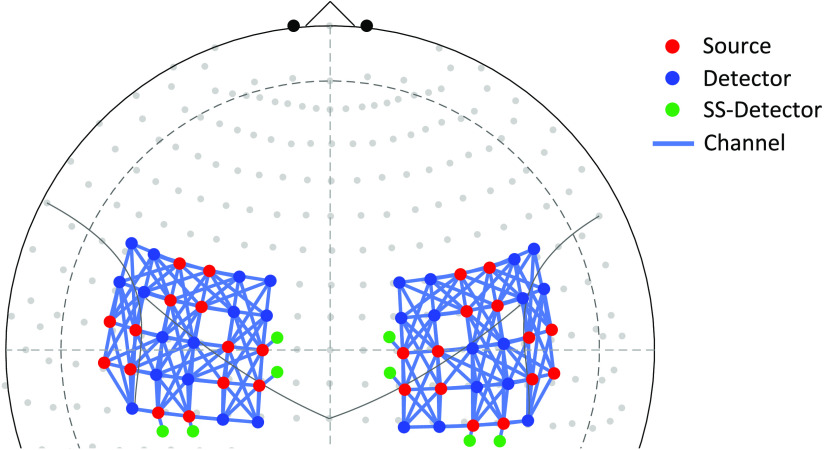
The high-density probe used in the experiment. A total of 219-channels (209 channels for long distance and eight channels for short-separation measurements) were distributed across bilateral motor and sensorimotor cortices. The distance between source and detector was 15 to 44 mm and 7.5 mm for long-distance and short-separation channels, respectively. Long-separation channels measure deeper cortical activity, whereas short-separation channels measure nonneuronal hemodynamic changes in skin (i.e., systemic physiological noise). Long-distance channels comprised 30 source optodes (red dots) and 31 detector optodes (blue dots) placed on the scalp. One detector optode split into eight detectors (green dots) was used for short-separation (SS) channels in eight locations across the probe. The light blue solid line represents the channels.

In this experiment, NIRS data were recorded using a commercial NIRScout-2 (NIRx, GmbH, Berlin, Germany) continuous fNIRS system with short-separation measurements. Figure 7 shows the configuration of the high-density probe used in this experiment, which includes a total of 219-channels (209 channels for long distance and eight channels for short-separation measurements) distributed across bilateral motor and sensorimotor cortices. The distance between source and detector was 15 to 44 mm and 7.5 mm for long-distance and short-separation channels, respectively. Long-separation channels measure deeper cortical activity, whereas short-separation channels measure nonneuronal hemodynamic changes in skin (i.e., systemic physiological noise). Long-distance channels comprised 30 source optodes (red dots) and 31 detector optodes (blue dots) placed on the scalp shown in Fig. 7. One detector optode split into 8 detectors (green dots) was used for short-separation channels in eight locations across the probe. The light blue solid line represents the channels. Data for two wavelengths (760 and 850 nm) were recorded at a sampling rate of 7.8125 Hz. After positioning the headcap, signal quality was optimized using the NIRx Aurora software. Ambient light was blocked using an opaque, black shower cap.

### 4.2 Subject and Task

Seven subjects participated in the experiment (six males, one female; age range 30 to 40 years; all right-handed). The subjects were informed about the experimentation and written consent was obtained. This study was provided by University of Pittsburgh Institutional Review Board.

**Fig. 8:**
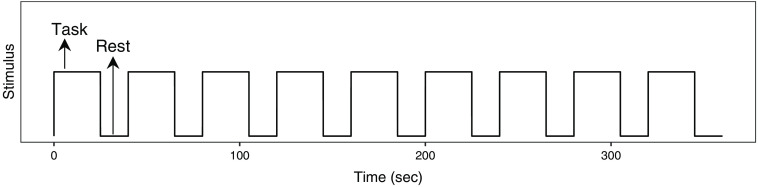
The time sequence of an experiment session. Each session (i.e., finger walking or sensory) consists of a 25 s task period followed by a 15 s rest period and was repeated nine times.

Each subject performed five scans consisting of one resting and four task scans (two sessions for both finger walking and sensory tasks). The subjects performed the experiment with their open eyes for both resting and task sessions. An experiment session (i.e., finger walking or sensory) consisted of a 25 s task period followed by a 15 s rest period and was repeated nine times as shown in Fig. 8.

The duration of the entire experiment was about 30 min. Subject were instructed on how to complete the paradigm. First, for the resting scan, the subjects were to avoid body motion and remain relaxed in the sitting position for 5 min without employing any mental effort. Next for the finger walking task, subjects were verbally instructed to do finger walking task. In the last, for sensory task, we used an electric brush to give the sensation sensory in the wrist. The five tasks were done in following order: resting-state, finger walking 1, finger walking 2, sensory 1, and sensory 2.

### 4.3 Expected ROIs

The expected ROIs for the finger walking and sensory tasks are the primary motor and somatosensory cortices. In BA pacellation, the primary motor cortex is BA-4, and the primary somatosensory consists of BA-3, BA-1, and BA-2. Thus, BA-3, BA-1, and BA-2 are regrouped into a larger group as the group constraint in the Ba-FSOGL model.

For the data collected from each task session, we run our Ba-FSOGL model following the steps described in Sec. 3 to obtain the image construction using this new approach. To compare this method to conventional image reconstruction, we also applied a previous published approach using error. In this part, we ReML model^3^ to the experimental data. The results from both methods are shown and compared in Sec. 5.4.

Considering the preknowledge of brain activation pattern is not always true, we also investigated the image reconstruction using a different errors. In this part, we reconstructed the images using BA-1/2/3 as prior for finger walking task, and BA-4 for sensory task.

## 5 Results

In this study, we ran the image reconstruction model 600 (simulation datasets) × 50 (initial values for $\lambda_{g}$) = 30,000 times in total. For the experimental data, we obtained four images (two finger walking and two sensories) for each subject. Each time the model costs approximately an hour to return the final result using MATLAB R2020a on macOS 10.15.6, Intel Core i7 2.6 GHz 6-core CPU, and 16 GByte memory. Since the time complexity of Gibbs sampling algorithm mainly depends on the model hierarchy and the number of sampling iterations, the time consumptions for simulation and experimental data are about the same. The entire over 30,000-h task was parallelly completed on a large-scale computer cluster. The results of the image reconstruction, statistical inference, and image evaluation are summarized in this section.

### 5.1 Reconstructed Image

**Fig. 9:**
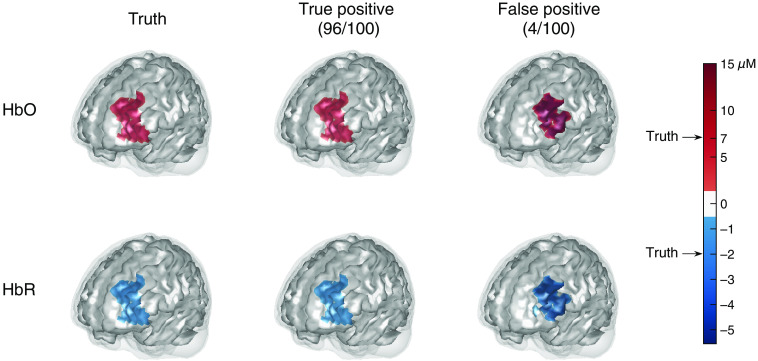
The ground truth and averaged reconstructed images for the datasets with activity in BA-46 left. The two rows indicate the images for HbO and HbR, respectively. The left column displays the two ground truth images whose colors are annotated on the color bar. The two images in the middle column are the averaged images that successfully recover a brain activity in BA-46 left (true positives). The two images in the right column are the averaged images that recover a brain activity in regions other than BA-46 left (false positives). In this case, 96 TPs and 4 FPs are obtained.

Figure 9 shows the truth and averaged reconstructed images for the datasets with BA-46 left active, respectively, which provide a visual comparison of the image reconstruction to the ground truth. In this figure, the two rows contain the images for HbO and HbR, respectively. The left column displays the two ground truth images whose colors are annotated on the color bar. The images in the remaining columns are the averaged reconstructed images where true and false positives are listed separately. The fraction under the column title of true/false positive indicates the proportion of successful/failed image reconstructions that are obtained to generate the averaged images. From Fig. 9, we can see that most of the datasets containing brain activity—96% for activity within BA-46 left–are successfully reconstructed as true positives, although the reconstructed activities are slightly smaller (lighter color) than the simulated truth. However, a small fraction of false positives can still be seen. The reconstructed images of activity in BA-10 and BA-45 left are similar to Fig. 9, so they are omitted here but shown in the Supplemental Material. Note that the color for the ground truth is preserved on the same color scale, i.e., 0, 7, and −2 are colored the same across the results figures, whereas the color scales for other values are different (see the color bar). Since BA-45 left and BA-46 left are at the same side of the probe and BA-45 left is further to the probe, the optical measurements for the activity within BA-45 left are sometimes similar to those for a smaller activity within BA-46 left, and vice versa, as explained in Sec. 1.3 of the Supplemental Material. Therefore, smaller activity (lighter color) in BA-46 left is reconstructed as FPs from 19% of the datasets containing activity within BA-45 left, and 4% false-positives are obtained from BA-46 left active datasets with larger brain activity (darker color) in BA-45 left.

**Fig. 10:**
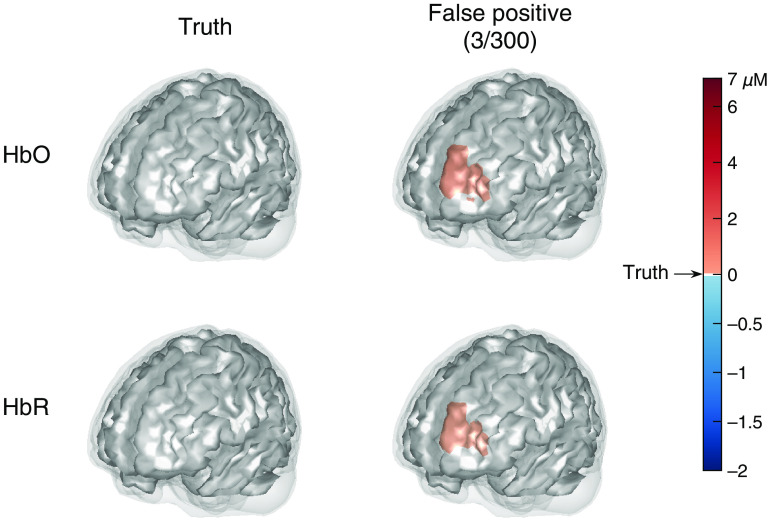
The ground truth and averaged reconstructed images for the datasets without brain activities. The two rows indicate the images for HbO and HbR, respectively. The left column displays the two ground truth images whose colors are annotated on the color bar. The two images in the right column are the averaged images that recover a brain activity in any region (false positives). In this case, three false positives out of 300 datasets are obtained.

Figure 10 shows the reconstructed images of the brain with no activated area (noise-only data). The plots demonstrate that our method only generates slight false positives within BA-10 left in 1% of the noise datasets.

### 5.2 Statistical Inference

**Fig. 11:**
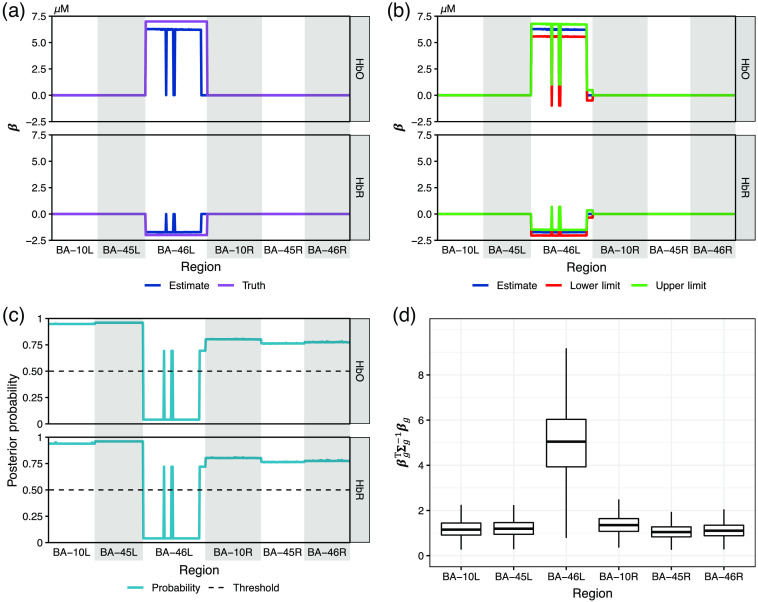
Four subplots showing the statistical inference for the image reconstruction of 100 datasets with brain activity simulated in BA-46 left. (a) The line plot of the ground truth and the estimated hemoglobin changes. (b) The estimated hemoglobin changes and the 50% CIs. (c) The posterior probability that $\beta_{p}$ is within the scaled neighborhood interval $\left[-\sqrt{\mathrm{var}(\beta_{p}|X,y)},\sqrt{\mathrm{var}(\beta_{p}|X,y)}\right]$ and the 50% probability threshold. (d) The boxplot $\beta_{g}^{T}\Sigma_{g}^{-1}\beta_{g}$ for all available BAs. Note that each point of the lines in (a)–(c) represents the value at a voxel belonging to the region indicated on the horizontal axis and is separated using the gray-shaded/white areas. Subplots (b)–(d) respectively show the statistical inference via the three approaches described in Sec. 3.2, from which we can conclude that the hemoglobin changes at most individual voxels in BA-46 left are significantly based on the CI and the probability within the scaled neighborhood interval, and the brain activity in BA-46 left is significantly larger than that in the remaining ROIs as an entirety.

**Fig. 12:**
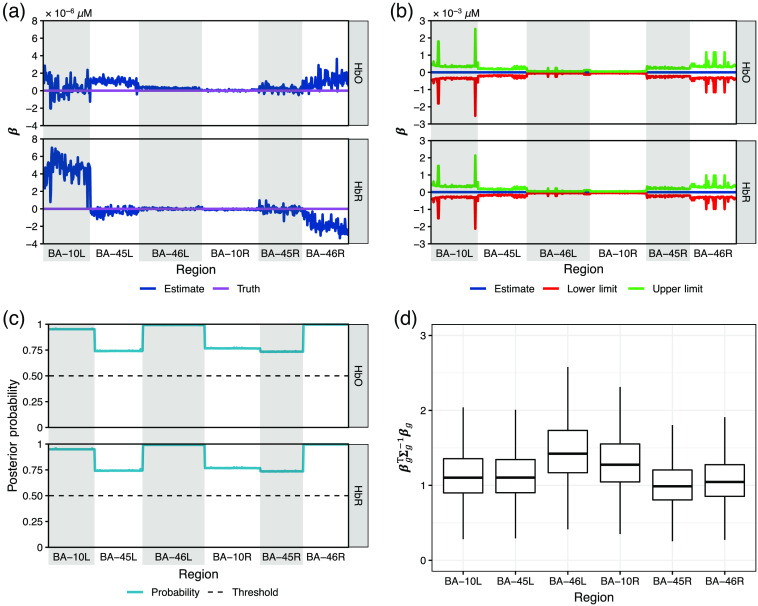
Four subplots showing the statistical inference for the image reconstruction of 100 datasets with no brain activity simulated in any areas. (a) The line plot of the ground truth and the estimated hemoglobin changes. (b) The estimated hemoglobin changes and the 50% CIs. (c) The posterior probability that $\beta_{p}$ is within the scaled neighborhood interval $\left[-\sqrt{\mathrm{var}(\beta_{p}|X,y)},\sqrt{\mathrm{var}(\beta_{p}|X,y)}\right]$ and the 50% probability threshold. (d) The boxplot $\beta_{g}^{T}\Sigma_{g}^{-1}\beta_{g}$ for all available BAs. Note that each point of the lines in (a)–(c) represents the value at a voxel belonging to the region indicated on the horizontal axis and separated using the gray-shaded/white areas. Sub-plots (b)–(d) respectively show the statistical inference via the three approaches described in Sec. 3.2, from which we can conclude that the hemoglobin changes at all individual voxels are insignificant based on the CI and the probability within the scaled neighborhood interval, and there is no brain activity in any ROI that is significantly larger than that in the remaining ROIs as an entirety.

Figure 11 shows the statistical inference results for the image reconstruction of datasets with brain activity simulated in BA-46 left. Each of the four figures consists of four subplots. The subplot in panel (a) is a line plot showing a clear comparison between the ground truth and the median of the estimates where we can see the absolute estimates for the voxels contained in active regions are slightly lower than the ground truths. Subplots (b)–(d) summarize the inference using the three methods described in Sec. 3.2, respectively. Note that each point on the lines of the truth, estimate, CI limit, and posterior probability in subplots (a)–(c) is calculated from the one million samples (10,000 samples/dataset × 100 datasets) for a specific HbO/HbR change at the voxel belonging to the area distinguished by the white/gray color and indicated at the x-axis, i.e., every point of the estimate line [dark blue in subplots (a) and (b)] represents the median, that of the lower/upper limit line [red/green line in subplot (b)] represents the lower/upper 50% quantile, and that of the posterior probability line [light blue line in subplot (c)] represents the fraction of samples within the scaled neighborhood interval. The boxplots in subplots (d) are calculated from the one million samples of $\beta_{g}^{T}\Sigma_{g}^{-1}\beta_{g}$ for the six regions. Figure 12 shows the statistical inference of the estimate for the noise datasets. Note that the number of samples used for generating [Figs. 12(a)–12(d)] is three million instead of one million used in Fig. 11 since there are 300 noise-only datasets. From Fig. 12(a), we can see that the estimates for the noise data fluctuate around the truths within a small range. The statistical inference results for the estimate of activities from BA-10 or BA-45 left are similar to Fig. 11 and consequently moved to the Supplemental Material.

From the four subplots of the statistical inference results for the activity within each ROI, we can see the three approaches for statistical inference described in Sec. 3.2 provide a consistent conclusion. It can be seen from subplots (b) that only the CI of the active areas exclude 0. In subplots (c), the posterior probability of the Gibbs samples within the scaled neighborhood interval is only below the 50% threshold for the active areas. Subplots (d) show that only the active areas have a nonoverlapping CI with the remaining areas. That is to say that statistical significance only appears in the truly active regions. Although there are a few exceptional voxels in active regions that do not show statistical significance (type-II error), we never see any statistical significance in any inactive regions (type-I error).

### 5.3 Image Evaluation

#### 5.3.1 Mean squared error and contrast-to-noise ratio

The results of MSE and CNR are summarized in Table 2. For each dataset, the MSE and CNR are calculated using Eqs. (6) and (7). The median of MSE and CNR of each 100 datasets with activity in BA-10, BA-45, and BA-46 left are shown in the table. Since CNR is not available for noise data, we only list the MSE median of the 300 noise datasets here. The reason we use median instead of mean of MSE and CNR here is that the FPs make remarkable detrimental contributions to the mean values although there are only a few FP cases. The values in this table indicate small discrepancies between the estimations and the simulation truths as well as large contrasts to distinguish the reconstructed brain activities from the background noise.

**Table 2:** The median of mean squared errors and the contrast-to-noise ratios (dB) of the HbO and HbR changes estimation for the datasets with different active regions.

	MSE median		CNR median (dB)	
Active region	HbO	HbR	HbO	HbR
BA-10 left	0.42	0.07	12.03	9.01
BA-45 left	0.55	0.11	10.42	7.29
BA-46 left	1.34	0.12	7.24	6.61
None (noise)	$1.84\times 10^{-10}$	$2.37\times 10^{-10}$	NA	NA

#### 5.3.2 ROC performance

**Fig. 13:**
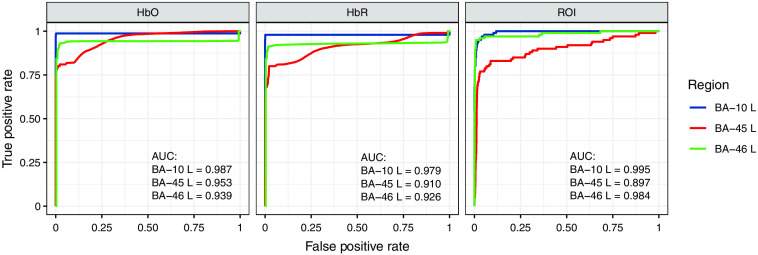
The ROI-ROC curves for the image reconstruction of the datasets with simulated activity in three different BAs against the corresponding noise data. The three active regions are indicated by the line color, and the two levels of ROC curve are indicated by the title of the three panels—two at voxel level (HbO and HbR) and one at ROI level. The large AUCs indicate the good ROC performance of the Ba-FSOGL model on fNIRS image reconstruction.

Figure 13 shows the ROI-ROC curves for the image reconstruction of the datasets with simulated activity in three different BAs against the corresponding noise data. The three active regions are indicated by the line color, and the two levels of ROC curve are indicated by the title of the three panels—two at voxel level (HbO and HbR) and one at ROI level. The AUCs of the ROC curves are shown at the lower-right corner of each panel. The AUC means the probability that the active voxels/regions have a higher rating than the inactive ones. As we can see, the AUCs are all >0.89, which indicates the good ROC performance of the Ba-FSOGL model on fNIRS image reconstruction.

**Fig. 14:**
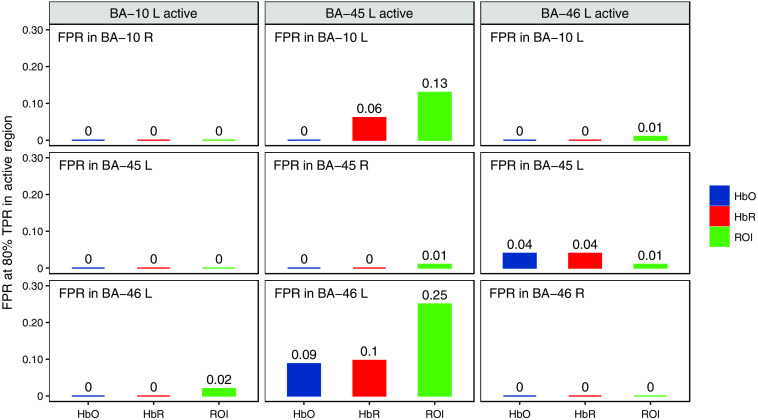
The bar chart showing the FPR in the region annotated at the up-left corner of each subplot when the TPR in the active region achieves 80%. The active region is indicated by the title of each column. The subplots on the main diagonal of the plot matrix show the FPR in the contralateral ROI whereas the remaining subplots show that in the neighboring ROIs. It can be seen that the FPRs in the contralateral ROIs are always smaller than those in the neighboring ROIs especially when BA-45 left is active.

In addition to the ROI–ROC performance, we also checked where FPs are easier to appear. Our hypothesis is that it is more common to see FPs in the neighboring regions next to the active region due to the low spatial resolution of fNIRS imaging. To test this hypothesis, we report the FPR in different regions when the TPR in the active region achieves 80% in Fig. 14, in which the subplots on the main diagonal of the plot matrix show the FPR in the contralateral ROI, whereas the remaining subplots show that in the neighboring ROIs. As we can see, the FPRs in the contralateral ROIs are always smaller than those in the neighboring ROIs especially when BA-45 left is active. Therefore, our hypothesis is valid.

### 5.4 Image Reconstruction for the Experimental Data

**Fig. 15:**
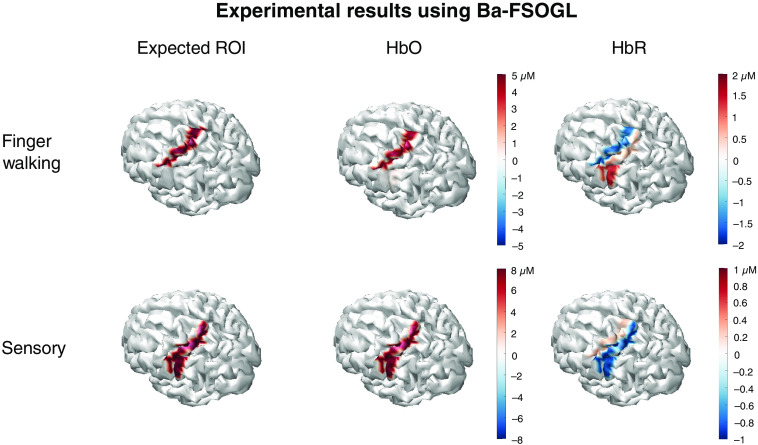
Image reconstruction results from the experimental data collected through the experiments described in Sec. 4 using the Ba-FSOGL model. In the finger walking and the sensory tasks, brain activities in the motor and somatosensory cortexes are expected, which are shown in the truth column. The HbO and HbR columns are reconstructed images for HbO and HbR changes. Each image is averaged over all the image reconstructions of subjects. Statistical inferences are also conducted using the methods described in Sec. 5.2. Insignificant changes are considered as noise and filtered out.

Figure 15 shows the image reconstruction results from the experimental data collected through the experiments described in Sec. 4 using the Ba-FSOGL model. In the finger walking and the sensory tasks, brain activities in the motor and somatosensory cortexes are expected, which are shown in the truth column of Fig. 15. The HbO and HbR columns are reconstructed images for HbO and HbR changes. Each image is averaged over all the image reconstructions of subjects. Statistical inferences are also conducted using the methods described in Sec. 5.2. Insignificant changes are considered as noise and filtered out.

From the results, it can be seen that our Ba-FSOGL model successfully reconstructed the two tasks. The two neighboring regions are distinguished by the image reconstruction, and the magnitudes of the hemoglobin change are in a reasonable range.

**Fig. 16:**
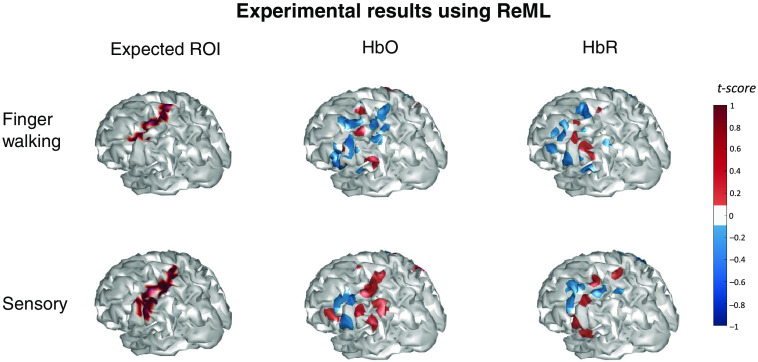
Image reconstruction results from the experimental data collected through the experiments described in Sec. 4 using the ReML model. The layout of Fig. 16 is similar to that of Fig. 15 except that the color scale indicates the t-scores reported by the model instead of the hemoglobin changes. Each image is also averaged over the image reconstructions of all subjects. The voxels with a t-score<0.1 are filtered out.

Figure 16 shows the image reconstruction results from the experimental data collected through the experiments described in Sec. 4 using the ReML model. The layout of Fig. 16 is similar to that of Fig. 15 except that the color scale indicates the t-scores reported by the model instead of the hemoglobin changes. Each image is also averaged over the image reconstructions of all subjects. The voxels with a t-score<0.1 are filtered out.

From the comparison of Figs. 15 and 16, we can see that although the ReML model successfully reconstructed brain activities around the expected ROIs, the reconstructed images are not as focal as those using Ba-FSOGL. It can also be noted that the statistical significances of the activities reconstructed using ReML are lower than using Ba-FSOGL as the maximum t-score around ±1 gives a p-value of 0.3.

**Fig. 17:**
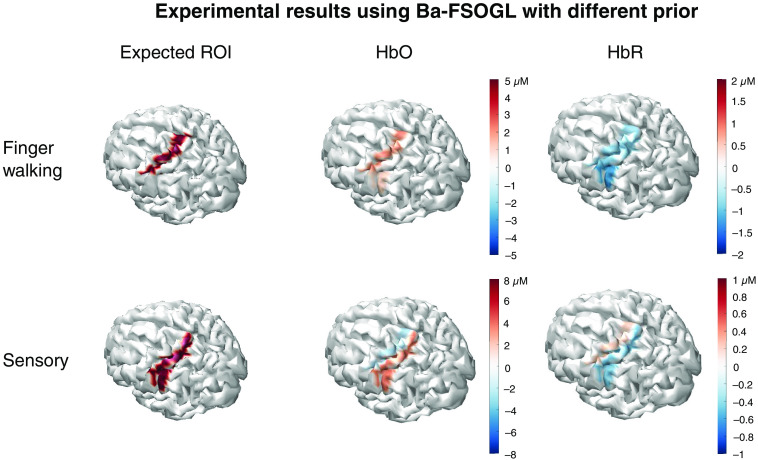
Shows the reconstructed images using Ba-FSOGL with BA-1/2/3 as prior for finger walking task, and BA-4 for sensory task. The layout of Fig. 17 is similar to that of Fig. 15. Each image is also averaged over the image reconstructions of all subjects. Statistical inferences are also conducted using the methods described in Sec. 5.2. Insignificant changes are considered as noise and filtered out. The color scales in Figs. 15 and 17 are the same for a convenient comparison.

Figure 17 shows the reconstructed images using Ba-FSOGL with BA-1/2/3 as prior for finger walking task and BA-4 for sensory task. The layout of Fig. 17 is similar to that of Fig. 15. Each image is also averaged over the image reconstructions of all subjects. Statistical inferences are also conducted using the methods described in Sec. 5.2. Insignificant changes are considered as noise and filtered out. The color scales in Figs. 15 and 17 are the same for a convenient comparison.

Comparing to Fig. 15, here we can see that the reconstructed activities are spread over the expected ROI and the prior area with lower magnitudes. This is reasonable because the data tends to suggest the activity in the expected ROI but the prior information suggests a different area. These two effects interact with each other, which results in the activities in both regions with a lower magnitude and statistical significance. The hyperparameter $\lambda_{g}$ is used to reflect the confidence of the prior information. If we are very sure the prior information is true, a large $\lambda_{g}$ can be set for that area.

## 6 Discussion

In this paper, we have described the proposed Ba-FSOGL model that involves anatomical and hemodynamics prior information in fNIRS image reconstruction and validated the model via numerical simulations. Now we will discuss the findings from the results in the following aspects.

### 6.1 Advantages of Ba-FSOGL

The motivation of the Ba-FSOGL algorithm is to place priors on the clustering of voxels by “lassoing” them based on the predefined underlying anatomical regions of interest. Therefore, voxels within the region of interest will have varied amplitude and spatial structure, but the effective model imposes that these voxels have a stronger relationship to each other (e.g., come from a common distribution) than they do to voxels outside this region. The result is that the boundaries of these ROIs are softly imposed on the edges of the reconstructed image. This allows statistical testing of both individual voxels within the image and the region-of-interest as a whole. A unique feature of this method is that these regions can overlap, which allows a voxel to belong to multiple groups in the LASSO algorithm.

From a mathematical perspective, the model proposed in this paper combines several common regularization terms. Each of them applies a type of constraint to the model based on the prior information. The fused lasso penalty minimizes the difference between neighboring connected coefficients. The group lasso term selects or excludes variables in the same group as much as possible and maintains the correlation between variables. The sparse term allows every individual variable in a group to be selected or excluded. The variable transformation of overlapping group lasso resolves the overlapping challenge by converting the problem into an equivalent regular minimization. From the results we show in Sec. 5, we can see that the anatomy and hemodynamics priors are all reflected in the reconstructed images. Thus, we can conclude the penalty terms we include in the proposed model are all appropriate and necessary. In addition, we use the adaptive version of regularization in this model, which allows different tuning parameters for groups. This is also an important feature will be discussed in Sec. 3.1. Finally, the model is solved in a Bayesian framework, which has several advantages over frequentist approaches. First, the samples from the Markov chain can be used for uncertainty estimation and statistical inference. Second, the optimization of the tuning parameters is integrated into the Gibbs sampling process. Third, it is fairly easy to incorporate the prior information into the model by involving multiple level latent variables. Finally, the hierarchical approach reduces the sensitivity of the latent variables to the measurement noise, especially in this high-dimensional inverse problem. Although the model’s hierarchy is enough to include the prior information of fNIRS image reconstruction, it is straightforward to extend the model for a more complex problem if necessary. For example, if the measurement noise cannot be easily decorrelated via whitening transformation, we can extend the model by replacing the identity matrix in Eq. (S2) with the noise covariance matrix and adding an extra level to model its pattern. Although we only validate this method using Brodmann parcellation as the anatomical prior, our model can actually handle different parcellations as long as the group membership of each β is reasonably determined. For instance, one may use the parcellation of the motor cortex according to the motor homunculus for a movement-involved experiment. Besides the anatomy and hemodynamic prior information considered in this paper, some other types of prior information can also be incorporated using this model. For example, taking the advantage of the adaptive tuning parameter, one may assign small penalty weight for the group representing the area that is expected to be active in the experiment, e.g., Broca’s area for speech- or language-related tasks.

To sum up, each penalty term of the proposed Ba-FSOGL model appropriately incorporates a type of prior information of fNIRS image reconstruction. The Bayesian algorithm allows statistical inference and provides extensionality.

### 6.2 Convergence of the Algorithm

**Fig. 18:**
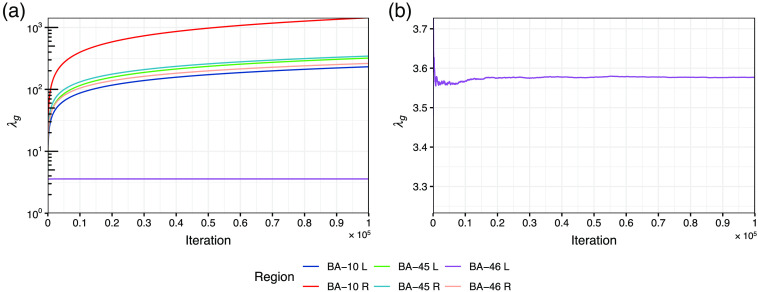
An example trace plot of $\lambda_{g}$ for a dataset containing brain activity in BA-46 left. (a) plot the value of tuning parameters for all regions (indicated by the line color) in the log scale as a function of sampling iteration. (b) Plot the value of the tuning parameter for the active region (BA-46 left in this example) in the original scale as a function of sampling iteration. It can be found from the plots that the tuning parameters of inactive regions increase as the sampling iteration while that of the active region fluctuates at the beginning and converges to a stable value at the end.

The convergence for the algorithm usually needs to be examined for MCMC-based approaches. Here, we show an example trace plot of $\lambda_{g}$ for a dataset containing brain activity in BA-46 left in Fig. 18.

It can be seen from the figure that the tuning parameter for the active region achieves a stable range while those for the inactive regions still increase at the end of the sampling chain. It looks diverging, however, the truth values of $\beta_{g}$ for inactive regions are zero. Thus, the diverging tuning parameter indicates the estimates converge to the truth. We examined all the trace plots and found they are all similar to Fig. 18. Therefore, we would consider the algorithm successfully converges. This also proves that the use of the adaptive regularization is necessary since it allows the tuning parameter for different regions to be different. Otherwise, the algorithm would be impossible to converge to the same results with an equal tuning parameter for all regions.

### 6.3 Missed Voxels

It can be clearly seen from Fig. 11 and Fig. S1 that the image reconstruction of datasets containing brain activity in BA-10 and BA-46 left have several false negatives where the estimates of the hemoglobin changes for some active voxels are insignificant. The two voxels missed in the BA-10 left image reconstruction can be seen in the brain space (Fig. S1), which indicates in the truth image that there are two voxels on a different gyrus. The two voxels are not connected to any other voxels in the spatial structure encoding matrix for BA-10 left. Since they are not connected to the main part of the region and are further from the probe than the main part, the regularization approach would tend to drop them as the estimates on them are larger but the difference between the main region is not constrained. The reason caused missed voxels in BA-46 left is the same, although they cannot be seen in the brain space (Fig. 9). The missed voxels are located on a layer under and not connected to the recovered part of BA-46 left either. Therefore, we can conclude that the missed voxels are caused by the anatomical prior information, and the algorithm does not have a problem.

### 6.4 Effects of Channel-space Prior

A question might be raised about the selection of the initial value of the tuning parameter. Since there is a possibility that the most active region indicated by channel-space ROI analysis is different from the truth, one may worry about that channel-space results mislead the image reconstruction model. In our simulation study using the 600 datasets, we also tried to provide the ground truth prior to the active region, which is impossible to know in a practical situation, to the image reconstruction model, however, the results do not change. In other words, it is impossible to correctly reconstruct the ground truth region from the datasets leading to false positives using this model regardless of the initial value of the tuning parameter. Thus, we can conclude that the prior information of active region provided by channel-space ROI analysis does not negatively affect the image reconstruction model.

### 6.5 Application to the Experimental Data

The results of the experimental data show us that our method not only performs well on the simulation data but also on real experimental data. The data were collected from human subjects using a high-density probe for different brain areas, which is very different from the simulation studies in terms of noise characteristics, montage type, active region position and size, and so on. The results demonstrate that our Ba-FSOGL model is adaptable to these situations.

### 6.6 Limitations and Future Plans

Although this paper demonstrates the good performance of the proposed image reconstruction model—Ba-FSOGL, there are still several limitations. First, the Gibbs sampling algorithm is time consuming. As we mentioned in Sec. 5, this work costs about 30,000 in total, which cannot be completed without a computer cluster. Second, we assume only one region is active in the datasets. Since it is challenging for the channel-space analysis to compare the significance in a small active region and a larger region containing a small active region, we make this assumption at this point. Third, unlike a frequentist approach, there is no p-value reported by the Bayesian model, so we cannot analyze the type-I error level of the model by comparing the empirical FPR to the type-I error control.

In addition, there are inherent limitations due to the low spatial resolution of fNIRS measurements. In particular, as shown in Fig. 14, this approach generates a moderate level of cross-talk into spatially neighboring regions of interest. For example, Fig. 14 shows about a 10% false-positive crosstalk for oxy-hemoglobin (25% for the ROI) in BA-46, when the true activity is simulated in the neighboring BA-45 region and threshold at 80% sensitivity for BA-45. This is not unexpected since in Talairach daemon atlas these two regions have a distance of only 16 mm between their region centers and come as close as 5 mm apart, which is below the expected resolution of the low-density fNIRS measurement probe used in this study.

Therefore, the next steps of this work will include implementing this model using a faster optimization algorithm, investigating on a more effective approach to determine the initial value of tuning parameter, and a frequentist approach for statistical inference.

## 7 Conclusion

We propose an approach for fNIRS image reconstruction by combining multiple lasso-based regularizations and solving the model in a Bayesian framework. The model is validated via numerical simulation and experimental data. The results of image reconstruction and statistical inference indicate the prior information on cerebral anatomy and hemodynamics is appropriately incorporated. The MSE, CNR, and ROC curves demonstrate the good performance of the model.

## Code, Data, and Materials Availability

Custom MATLAB scripts, anonymized data and other materials used in this study can be made available for interested parties upon request.
