# Supplementary material for: Brain space image reconstruction of functional near-infrared spectroscopy using a Bayesian adaptive fused sparse overlapping group lasso model
Source: Neurophotonics. 2023 Feb 10;10(2):023516. doi: 10.1117/1.NPh.10.2.023516 (PMC9912979; doi:10.1117/1.NPh.10.2.023516)
Supplement: Supplementary file 1 [file NPh_010_023516_SD001.pdf]

# Brain space image reconstruction of functional near-infrared spectroscopy (fNIRS) using a novel Bayesian adaptive fused sparse overlapping group lasso (Ba-FSOGL) model

**Xuetong Zhai,[1] Hendrik Santosa,[2] Robert T. Krafty,[3] Theodore J. Huppert[4,*]**

[1]*University of Pittsburgh, Department of Electrical and Computer Engineering, Pittsburgh, Pennsylvania 15213, United States*
[2]*University of Pittsburgh, Department of Radiology, Pittsburgh, Pennsylvania 15213, United States*
[3]*Emory University, Department of Biostatistics and Bioinformatics, Atlanta, Georgia, 30322, United States*
[4]*University of Pittsburgh, Department of Electrical and Computer Engineering, Department of Bioengineering, Clinical Science Translational Institute, and Center for the Neural Basis of Cognition, Pittsburgh, Pennsylvania 15213, United States*
**huppert1@pitt.edu*

## 1. Details of the Bayesian Modeling

### *1.1 Bayesian hierarchical modeling and prior distributions*

The number of parameters need to be optimized in a-FSOGL is usually more than 1,000 including $\boldsymbol{\beta}$ and its covariance matrix. Searching in such a high-dimensional solution space and maintaining the semipositive definiteness of the covariance matrix are challenging using the conventional gradient-based minimization algorithms. Alternativly, penalized least squares estimators of the form of Eq (4) have an alternative interpretation as the Bayes posterior mode under a suitiably selected heirarical model. In this subsection, we propose the hierarchical Bayesian a-FSOGL (Ba-FSOGL) based on the contributions of previous studies [12, 13, 15, 16] and extend it to handle correlated coefficients by involving the covariance matrix.

Similar to previous studies [12], the conditional prior of $\boldsymbol{\beta}$ for the model in Eq. (5) of the manuscript can be written as Eq.(S1).

$$\pi\left(\boldsymbol{\beta}|\sigma^2,\mathbf{C}_{\boldsymbol{\beta}_g},\mathbf{D}_g\right)\propto\exp\left\{\frac{1}{\sigma}\sum_{g=1}^{G}\lambda_g\left[\theta\gamma\left\|\boldsymbol{\beta}_g\right\|_1+(1-\gamma)\left\|\mathbf{D}_g\boldsymbol{\beta}_g\right\|_1+(1-\theta)\gamma\left\|\boldsymbol{\beta}_g\right\|_{\mathbf{C}_{\boldsymbol{\beta}_g}^{-1}}\right]\right\}\tag{S1}$$

We introduce the hierarchical model with the latent parameters:

$$\mathbf{y}|\mathbf{X},\boldsymbol{\beta},\sigma^2\sim\mathcal{N}_N(\mathbf{X}\boldsymbol{\beta},\sigma^2\mathbf{I})\tag{S2}$$

where $\mathcal{N}_n(\boldsymbol{\mu},\boldsymbol{\Sigma})$ denotes a $n$-dimensional multivariate normal distribution with a mean vector $\boldsymbol{\mu}$ and a covariance matrix $\boldsymbol{\Sigma}$, and $\sigma^2$ is the noise variance in the measurement space. For the $g$-th group, we introduce the prior distribution of $\boldsymbol{\beta}_g$ as follows.

$$\boldsymbol{\beta}_g|\mathbf{X},\boldsymbol{\Sigma}_g,\sigma^2\sim\mathcal{N}_{m_g}\left(\mathbf{0},\sigma^2\boldsymbol{\Sigma}_g\right)\tag{S3}$$

The inverse of the covariance matrix $\boldsymbol{\Sigma}_g$ should have the following structure reflecting the constrains from the three penalty terms in the model from Eq. (5).

$$\boldsymbol{\Sigma}_g=\left[\boldsymbol{\Psi}_g^{-1}+\mathbf{D}_g^{\mathrm{T}}\boldsymbol{\Phi}_g^{-1}\mathbf{D}_g+\left(\tau_g^2\mathbf{C}_{\boldsymbol{\beta}_g}\right)^{-1}\right]^{-1}\tag{S4}$$

The $\boldsymbol{\Psi}_g$matrix defines the variance of each element of $\boldsymbol{\beta}_g$, which allows variable selection at individual level with the following structure.

$$\mathbf{\Psi}_g = \begin{bmatrix} \psi_{g,1}^2 & 0 & \cdots & 0 \\ 0 & \psi_{g,2}^2 & \ddots & 0 \\ \vdots & \ddots & \ddots & \vdots \\ 0 & 0 & \cdots & \psi_{g,m_g}^2 \end{bmatrix} \tag{S5}$$

The matrix $\mathbf{D}_g^{\mathrm{T}}\mathbf{\Phi}_g^{-1}\mathbf{D}_g$ incorporates the effect of fused lasso into the model where

$$\mathbf{\Phi}_g = \begin{bmatrix} \phi_{g,1}^2 & 0 & \cdots & 0 \\ 0 & \phi_{g,2}^2 & \ddots & 0 \\ \vdots & \ddots & \ddots & \vdots \\ 0 & 0 & \cdots & \phi_{g,q_g}^2 \end{bmatrix} \tag{S6}$$

The matrix $\tau_g^2\mathbf{C}_{\boldsymbol{\beta}_g}$ holds the group level variable selection where $\tau_g^2$ is the common variance term of $\boldsymbol{\beta}_g$ and $\mathbf{C}_{\boldsymbol{\beta}_g}$ is a semi-positive definite matrix constraining the relationship between the elements among $\boldsymbol{\beta}_g$. In the case of fNIRS image reconstruction, the first half elements of $\boldsymbol{\beta}_g$ represents the HbO and the other half represents the HbR changes within a region. Hence, considering the common variance has been determined by $\tau_g^2$, $\mathbf{C}_{\boldsymbol{\beta}_g}$ only needs to reflect the anticorrelation between HbO and HbR changes as well as the fraction of HbR changes to HbO changes. Thus, we define $\mathbf{C}_{\boldsymbol{\beta}_g}$ as a $m_g \times m_g$ matrix.

$$\mathbf{C}_{\boldsymbol{\beta}_g} = \begin{bmatrix} 1 & \rho_g\zeta_g \\ \rho_g\zeta_g & \zeta_g^2 \end{bmatrix} \otimes \mathbf{I}_{m_g/2}$$

$$\rho_g \sim \mathcal{U}(-1, 0) \text{ and } \zeta_g \sim \mathcal{U}(0,1) \tag{S7}$$

where $\otimes$ stands for Kronecker product and $\mathcal{U}(a, b)$ denotes a uniform distribution between $[a, b]$. The $-1 \leq \rho_g \leq 0$ ensures the negative correlation between HbO and HbR changes, and $0 \leq \zeta_g \leq 1$ maintains the amplitude of the HbR change is smaller than that of HbO change at the same voxel.

We place the following multivariate prior on $\psi_{g,1}^2, \psi_{g,2}^2 \cdots \psi_{g,m_g}^2, \phi_{g,1}^2, \phi_{g,2}^2 \cdots \phi_{g,q_g}^2, \tau_g^2$:

$$\pi\left(\psi_{g,1}^2, \cdots \psi_{g,m_g}^2, \phi_{g,1}^2, \cdots \phi_{g,k_g}^2, \tau_g^2\right)$$

$$= \text{constant} \cdot \det(\mathbf{\Sigma}_g)^{\frac{1}{2}} \cdot \prod_{p=1}^{m_g} \left\{ \left(\psi_{g,p}^2\right)^{-\frac{1}{2}} \frac{\left(\lambda_g\theta\gamma\right)^2}{2} \exp\left[-\frac{\left(\lambda_g\theta\gamma\right)^2 \psi_{g,p}^2}{2}\right] \right\}$$

$$\cdot \prod_{k=1}^{q_g} \left( \left(\phi_{g,k}^2\right)^{-\frac{1}{2}} \frac{\left[\lambda_g(1-\gamma)\right]^2}{2} \exp\left\{-\frac{\left[\lambda_g(1-\gamma)\right]^2 \phi_{g,k}^2}{2}\right\} \right)$$

$$\cdot \left(\tau_g^2\right)^{-\frac{1}{2}} \frac{\left[\lambda_g(1-\theta)\gamma\right]^2}{2} \exp\left\{-\frac{\left[\lambda_g(1-\theta)\gamma\right]^2 \tau_g^2}{2}\right\} \tag{S8}$$

For each $g = 1, \dots, G$, similar to Eq. (16) in [12], the marginal distribution of $\boldsymbol{\beta}_g$ can be drived by Eq. (S9). The number below the ellipsis in Eq. (S9) denotes the number of integral operators omitted.

$$
\int_0^{+\infty} \underset{m_g-1}{\cdots\cdots} \int_0^{+\infty} \underset{q_g-1}{\cdots\cdots} \int_0^{+\infty} \left[ \pi(\boldsymbol{\beta}_g | \mathbf{X}, \boldsymbol{\Sigma}_g, \sigma^2) \pi\left(\psi_{g,1}^2, \cdots \psi_{g,m_g}^2, \phi_{g,1}^2, \cdots \phi_{g,q_g}^2, \tau_g^2\right) \cdot \prod_{p=1}^{m_g} \mathrm{d}\psi_{g,p}^2 \prod_{k=1}^{q_g} \mathrm{d}\phi_{g,k}^2 \, \mathrm{d}\tau_g^2 \right]
$$

$$
\propto \int_0^{+\infty} \underset{m_g-1}{\cdots\cdots} \int_0^{+\infty} \underset{q_g-1}{\cdots\cdots} \int_0^{+\infty} \left\{ \begin{array}{c} \det(\boldsymbol{\Sigma}_g)^{-\frac{1}{2}} \exp\left[-\frac{1}{2} \boldsymbol{\beta}_g^{\mathrm{T}} (\sigma^2 \boldsymbol{\Sigma}_g)^{-1} \boldsymbol{\beta}_g\right] \\ \cdot \det(\boldsymbol{\Sigma}_g)^{\frac{1}{2}} \prod_{p=1}^{m_g} \left\{ (\psi_{g,p}^2)^{-\frac{1}{2}} \frac{(\lambda_g \theta \gamma)^2}{2} \exp\left[-\frac{(\lambda_g \theta \gamma)^2 \psi_{g,p}^2}{2}\right] \right\} \\ \cdot \prod_{k=1}^{q_g} \left( (\phi_{g,k}^2)^{-\frac{1}{2}} \frac{[\lambda_g(1-\gamma)]^2}{2} \exp\left\{ -\frac{[\lambda_g(1-\gamma)]^2 \phi_{g,k}^2}{2} \right\} \right) \\ \cdot (\tau_g^2)^{-\frac{1}{2}} \frac{[\lambda_g(1-\theta)\gamma]^2}{2} \exp\left\{ -\frac{[\lambda_g(1-\theta)\gamma]^2 \tau_g^2}{2} \right\} \\ \cdot \prod_{p=1}^{m_g} \mathrm{d}\psi_{g,p}^2 \prod_{k=1}^{q_g} \mathrm{d}\phi_{g,k}^2 \, \mathrm{d}\tau_g^2 \end{array} \right\}
$$

$$
\propto \int_0^{+\infty} \underset{m_g-2}{\cdots\cdots} \int_0^{+\infty} \exp\left(-\frac{\boldsymbol{\beta}_g^{\mathrm{T}} \boldsymbol{\Psi}_g^{-1} \boldsymbol{\beta}_g}{2\sigma^2}\right) \prod_{p=1}^{m_g} \left\{ (\psi_{g,p}^2)^{-\frac{1}{2}} \frac{(\lambda_g \theta \gamma)^2}{2} \exp\left[-\frac{(\lambda_g \theta \gamma)^2 \psi_{g,p}^2}{2}\right] \mathrm{d}\psi_{g,p}^2 \right\}
$$

$$
\cdot \int_0^{+\infty} \underset{q_g-2}{\cdots\cdots} \int_0^{+\infty} \exp\left(-\frac{\boldsymbol{\beta}_g^{\mathrm{T}} \mathbf{D}_g^{\mathrm{T}} \boldsymbol{\Phi}_g^{-1} \mathbf{D}_g \boldsymbol{\beta}_g}{2\sigma^2}\right)
$$

$$
\cdot \prod_{k=1}^{q_g} \left( (\phi_{g,k}^2)^{-\frac{1}{2}} \frac{[\lambda_g(1-\gamma)]^2}{2} \exp\left\{ -\frac{[\lambda_g(1-\gamma)]^2 \phi_{g,k}^2}{2} \right\} \mathrm{d}\phi_{g,k}^2 \right)
$$

$$
\cdot \int_0^{+\infty} \exp\left(-\frac{\boldsymbol{\beta}_g^{\mathrm{T}} \mathbf{C}_{\boldsymbol{\beta}_g}^{-1} \boldsymbol{\beta}_g}{2\sigma^2 \tau_g^2}\right) (\tau_g^2)^{-\frac{1}{2}} \frac{[\lambda_g(1-\theta)\gamma]^2}{2} \exp\left\{ -\frac{[\lambda_g(1-\theta)\gamma]^2 \tau_g^2}{2} \right\} \mathrm{d}\tau_g^2
$$

$$
\propto \exp\left\{ \frac{\lambda_g}{\sigma} \left[ \theta\gamma \|\boldsymbol{\beta}_g\|_1 + (1-\gamma) \|\mathbf{D}_g \boldsymbol{\beta}_g\|_1 + (1-\theta)\gamma \|\boldsymbol{\beta}_g\|_{\mathbf{C}_{\boldsymbol{\beta}_g}^{-1}} \right] \right\} \tag{S9}
$$

where $\pi(\boldsymbol{\beta}_g)$ is the probability density function of the prior distribution of $\boldsymbol{\beta}_g$ defined by Eq. (S3). The last step of Eq. (S9) is based on Eq. (S10), which demonstrates the double-exponential (Laplace) distribution is a scale mixture of a Gaussian distribution with an exponential density.

$$
\frac{a}{2} \exp(-a|z|) = \int_0^{\infty} \frac{1}{\sqrt{2\pi t}} \exp\left(-\frac{z^2}{2t}\right) \frac{a^2}{2} \exp\left(-\frac{a^2}{2} t\right) \mathrm{d}t \tag{S10}
$$

The conditional prior can be calculated by the product of Eq. (S9) for $g = 1$ through $G$ given by Eq. (S11), which satisfies the conditional prior of $\boldsymbol{\beta}$ anticipated in Eq. (S1).

$$\prod_{g=1}^{G} \exp\left\{\frac{\lambda_g}{\sigma}\left[\theta\gamma\|\boldsymbol{\beta}_g\|_1 + (1-\gamma)\|\mathbf{D}_g\boldsymbol{\beta}_g\|_1 + (1-\theta)\gamma\|\boldsymbol{\beta}_g\|_{\mathbf{C}_{\boldsymbol{\beta}_g}^{-1}}\right]\right\}$$
$$= \exp\left\{\frac{1}{\sigma}\sum_{g=1}^{G}\lambda_g\left[\theta\gamma\|\boldsymbol{\beta}_g\|_1 + (1-\gamma)\|\mathbf{D}_g\boldsymbol{\beta}_g\|_1 + (1-\theta)\gamma\|\boldsymbol{\beta}_g\|_{\mathbf{C}_{\boldsymbol{\beta}_g}^{-1}}\right]\right\} \quad \text{(S11)}$$

## 1.2 Gibbs sampling from full conditional distributions

With the hierarchical model described in Sec. 1.1, $\boldsymbol{\beta}_g$ can be estimated using its empirical posterior distribution obtained by Gibbs sampling, which requires the full conditional distribution – the posterior distribution depending on all remaining parameters – of every model parameter. This section will show the steps for calculating the full conditional distributions.

Similar to previous studies, we can interpose an inverse gamma ($\mathcal{IG}$) hyperprior for $\sigma^2$ in addition to the prior distributions given in Sec. 1.1 defined in Eq. (S12).

$$\sigma^2 \sim \mathcal{IG}(r, s) \quad \text{(S12)}$$

where $r$ and $s$ are the shape and scale hyperparameter of inverse gamma distribution. The joint posterior probability density function (PDF) of $\boldsymbol{\beta}, \psi^2, \phi^2, \tau^2, \rho, \zeta$ given $\mathbf{X}, \mathbf{y}$ is shown in Eq. (S13).

$$\pi(\boldsymbol{\beta}, \psi^2, \phi^2, \tau^2, \rho, \zeta | \mathbf{X}, \mathbf{y})$$
$$= \pi(\mathbf{y}|\mathbf{X}, \boldsymbol{\beta}, \sigma^2)\pi(\sigma^2)\prod_{g=1}^{G}\left[\pi(\boldsymbol{\beta}_g|\mathbf{X}, \boldsymbol{\Sigma}_g, \sigma^2)\pi\left(\psi_{g,1}^2, \cdots \psi_{g,m_g}^2, \phi_{g,1}^2, \cdots \phi_{g,q_g}^2, \tau_g^2\right)\pi(\zeta_g)\pi(\rho_g)\right]$$
$$= (2\pi\sigma^2)^{-\frac{N}{2}}\exp\left[-\frac{(\mathbf{y}-\mathbf{X}\boldsymbol{\beta})^{\mathrm{T}}(\mathbf{y}-\mathbf{X}\boldsymbol{\beta})}{2\sigma^2}\right]\cdot\frac{s^r(\sigma^2)^{-r-1}}{\Gamma(r)}\exp\left(-\frac{s}{\sigma^2}\right)$$
$$\cdot\prod_{g=1}^{G}\left\{(2\pi\sigma^2)^{\frac{m_g}{2}}\det(\boldsymbol{\Sigma}_g)^{-\frac{1}{2}}\cdot\exp\left(-\frac{1}{2\sigma^2}\boldsymbol{\beta}_g^{\mathrm{T}}\boldsymbol{\Sigma}_g^{-1}\boldsymbol{\beta}_g\right)\cdot\text{constant}\cdot\det(\boldsymbol{\Sigma}_g)^{\frac{1}{2}}\right.$$
$$\cdot\prod_{p=1}^{m_g}\left\{(\psi_{g,p}^2)^{-\frac{1}{2}}\frac{(\lambda_g\theta\gamma)^2}{2}\exp\left[-\frac{(\lambda_g\theta\gamma)^2\psi_{g,p}^2}{2}\right]\right\}$$
$$\cdot\prod_{k=1}^{q_g}\left((\phi_{g,k}^2)^{-\frac{1}{2}}\frac{[\lambda_g(1-\gamma)]^2}{2}\exp\left\{-\frac{[\lambda_g(1-\gamma)]^2\phi_{g,k}^2}{2}\right\}\right)$$
$$\left.\cdot(\tau_g^2)^{-\frac{1}{2}}\frac{[\lambda_g(1-\theta)\gamma]^2}{2}\exp\left\{-\frac{[\lambda_g(1-\theta)\gamma]^2\tau_g^2}{2}\right\}\cdot 1\cdot 1\right\} \quad \text{(S13)}$$

Let $\mathbf{A}_g = \mathbf{X}_g^{\mathrm{T}}\mathbf{X}_g + \boldsymbol{\Sigma}_g^{-1}$ where $\mathbf{X}_g$ is a submatrix of $\mathbf{X}$ containing the columns corresponding to $\boldsymbol{\beta}_g$. The terms involving $\boldsymbol{\beta}_g$ can be written as Eq. (S14), which is proportional to the PDF of a multivariate normal distribution.

$$\exp\left\{-\frac{1}{2\sigma^2}\left[\boldsymbol{\beta}_g - \mathbf{A}_g^{-1}\mathbf{X}_g^{\mathrm{T}}\left(\mathbf{y}-\sum_{g'\neq g}\mathbf{X}_{g'}\boldsymbol{\beta}_{g'}\right)\right]^{\mathrm{T}}\mathbf{A}_g\left[\boldsymbol{\beta}_g - \mathbf{A}_g^{-1}\mathbf{X}_g^{\mathrm{T}}\left(\mathbf{y}-\sum_{g'\neq g}\mathbf{X}_{g'}\boldsymbol{\beta}_{g'}\right)\right]\right\} \quad \text{(S14)}$$

The full conditional posterior of $\boldsymbol{\beta}_g$ is therefore

$$\boldsymbol{\beta}_g|\text{rest}\sim\mathcal{N}_{m_g}\left(\mathbf{A}_g^{-1}\mathbf{X}_g^{\mathrm{T}}\left(\mathbf{y}-\sum_{g'\neq g}\mathbf{X}_{g'}\boldsymbol{\beta}_{g'}\right),\sigma^2\mathbf{A}_g^{-1}\right) \tag{S15}$$

where rest represents all the remaining parameters. Following similar steps, we can derive the full conditional posterior of the other parameters shown as follows.

$$\frac{1}{\psi_{g,p}^2}|\text{rest}\overset{\text{ind.}}{\sim}i\mathcal{G}\left(\sqrt{\frac{\left(\lambda_g\theta\gamma\right)^2\sigma^2}{\beta_{g,p}^2}},\left(\lambda_g\theta\gamma\right)^2\right) \tag{S16}$$

$$g=1\dots G;p=1\dots m_g$$

$$\frac{1}{\phi_{g,k}^2}|\text{rest}\overset{\text{ind.}}{\sim}i\mathcal{G}\left(\sqrt{\frac{\left[\lambda_g(1-\gamma)\right]^2\sigma^2}{\left(\mathbf{D}_g\boldsymbol{\beta}_g\right)_k^2}},\left[\lambda_g(1-\gamma)\right]^2\right) \tag{S17}$$

$$g=1\dots G;k=1\dots q_g$$

$$\frac{1}{\tau_g^2}|\text{rest}\overset{\text{ind.}}{\sim}i\mathcal{G}\left(\sqrt{\frac{\left[\lambda_g(1-\theta)\gamma\right]^2\sigma^2}{\left\|\boldsymbol{\beta}_g\right\|_{\mathbf{C}_{\boldsymbol{\beta}_g}^{-1}}^2}},\left[\lambda_g(1-\theta)\gamma\right]^2\right) \tag{S18}$$

$$g=1\dots G$$

$$\sigma^2|\,\text{rest}\sim i\mathcal{g}\left(\frac{N-1+P}{2}+r,\frac{1}{2}\|\mathbf{y}-\mathbf{X}\boldsymbol{\beta}\|^2+\frac{1}{2}\sum_{g=1}^{G}\left\|\boldsymbol{\beta}_g\right\|_{\boldsymbol{\Sigma}_g^{-1}}^2+s\right) \tag{S19}$$

where $i\mathcal{G}(\mu,\upsilon)$ denotes an inverse Gaussian distribution with mean $\mu$ and scale parameters $\upsilon$.

Let $\beta_{g,1}^{\text{HbO}}\cdots\beta_{g,m_g/2}^{\text{HbO}}$ and $\beta_{g,1}^{\text{HbR}}\cdots\beta_{g,m_g/2}^{\text{HbR}}$ respectively denote the HbO and HbR changes at the $\frac{m_g}{2}$ positions in the $g$-th group. The term involving $\mathbf{C}_{\boldsymbol{\beta}_g}$ can be expanded as Eq. (S20).

$$\exp\left[-\frac{1}{2}\boldsymbol{\beta}_g^{\mathrm{T}}\left(\sigma^2\tau_g^2\mathbf{C}_{\boldsymbol{\beta}_g}\right)^{-1}\boldsymbol{\beta}_g\right]$$
$$=\exp\left(-\frac{1}{2\left(1-\rho_g^2\right)\sigma^2\tau_g^2}\left\{\sum_{p=1}^{m_g/2}\left[\left(\beta_{g,p}^{\text{HbO}}\right)^2-\frac{2\rho_g\beta_{g,p}^{\text{HbO}}\beta_{g,p}^{\text{HbR}}}{\zeta_g}+\frac{\left(\beta_{g,p}^{\text{HbR}}\right)^2}{\zeta_g^2}\right]\right\}\right) \tag{S20}$$

The full conditionals for $\zeta_g$ and $\rho_g$ are respectively proportional to Eq. (S21) and Eq. (S22).

$$\exp\left(-\frac{1}{2\left(1-\rho_g^2\right)\sigma^2\tau_g^2}\left\{\sum_{p=1}^{m_g/2}\left[-\frac{2\rho_g\beta_{g,p}^{\text{HbO}}\beta_{g,p}^{\text{HbR}}}{\zeta_g}+\frac{\left(\beta_{g,p}^{\text{HbR}}\right)^2}{\zeta_g^2}\right]\right\}\right) \tag{S21}$$

$$\exp\left(-\frac{1}{2\left(1-\rho_g^2\right)\sigma^2\tau_g^2}\left\{\sum_{p=1}^{m_g/2}\left[\left(\beta_{g,p}^{\text{HbO}}\right)^2-\frac{2\rho_g\beta_{g,p}^{\text{HbO}}\beta_{g,p}^{\text{HbR}}}{\zeta_g}+\frac{\left(\beta_{g,p}^{\text{HbR}}\right)^2}{\zeta_g^2}\right]\right\}\right) \tag{S22}$$

With the full conditional distributions of the parameters in the model, one can estimate the marginal distribution of $\boldsymbol{\beta}$ using a Markov chain Monte Carlo (MCMC) method – Gibbs sampler. In each sampling iteration, every parameter is sampled from its full conditional distribution using the values of the remaining parameters sampled from the previous iteration. Note that full conditionals of $\rho,\zeta$ are not known distributions, which cannot be sampled directly. We plug in Metropolis-Hastings sampler within each Gibbs sampling iteration to obtain the

samples of $\rho, \zeta$ [54]. After the sampling chain converges, $\boldsymbol{\beta}$ can be estimated by the mean or median of its samples.

### 1.3 Choosing the tuning parameters

The tuning parameter $\lambda_g$ determins the level of regularization. In this study, we choose the tuning parameter using a stochastic approximation-based single-step approach proposed by previous studies [13, 55] for a given dataset $\mathbf{X}, \mathbf{y}$, which is a computationally economical single-step approach.

In the $i$-th sampling iteration of our framework, transforming the tuning parameter by $\delta_g = \log \lambda_g$, we update $\delta_g$ following the rule $\delta_g^{(i)} = \delta_g^{(i-1)} + u_j \cdot \left(\frac{\partial \mathcal{LL}}{\partial \delta_g}\right)$ where $\mathcal{LL}$ is the log-likelihood function of $\lambda_g$ and $\{u_i\}$ is a positive sequence satisfying the following conditions:

1. $u_i$ monotonically decreases and converges to 0 as $i$ increases;
2. $\sum_{i=1}^{\infty} u_i = \infty$;
3. $\sum_{i=1}^{\infty} u_i^2 < \infty$.

In the simulation study of this paper, $u_i$ is set to be the terms of a scaled harmonic series $u_i = \frac{10^{-3}}{i}$. The scaling factor, $10^{-3}$, determines the optimization step size selected by preliminary trials. We need to find a moderate value, with which the algorithm coverges within reasonable iterations time without diverging.

## 2. Supplementary Results Figures

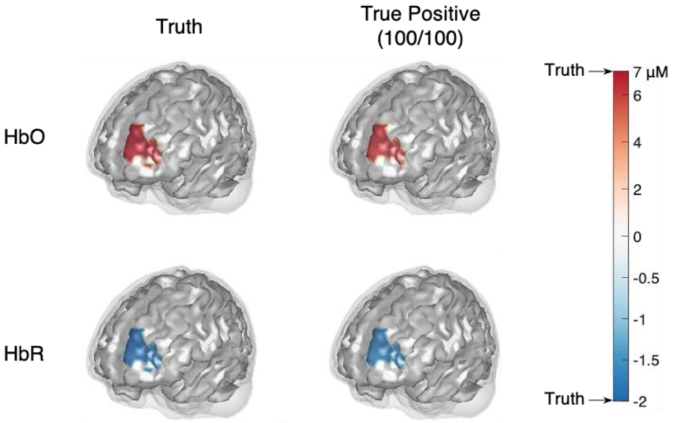


Fig. S1. The ground truth and averaged reconstructed image for the datasets with activity in BA-10 left. The two rows indicate the images for HbO and HbR respectively. The left column displays the two ground truth images whose colors are annotated on the color bar. The two images in the right column are the averaged images that successfully recover a brain activity in BA-10 left (true positives). In this case, all 100 datasets are successfully recovered with a slightly smaller activity.

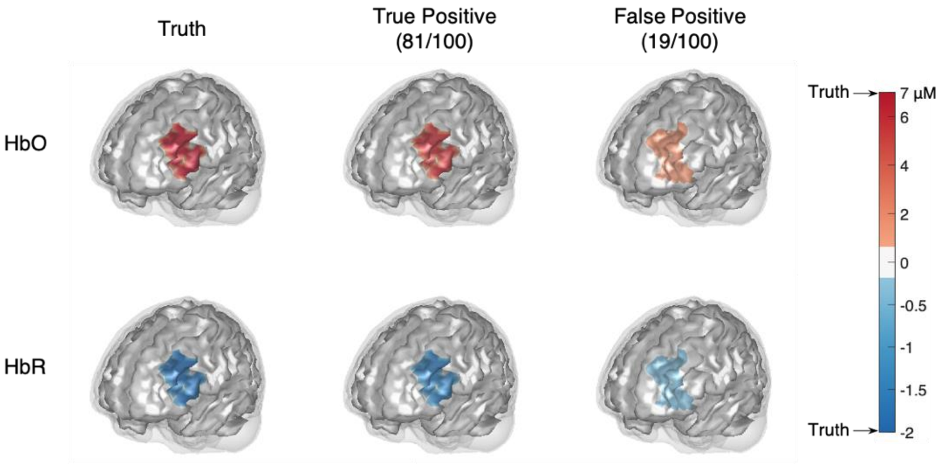


Fig. S2. The ground truth and averaged reconstructed images for the datasets with activity in BA-45 left. The two rows indicate the images for HbO and HbR respectively. The left column displays the two ground truth images whose colors are annotated on the color bar. The two images in the middle column are the averaged images that successfully recover a brain activity in BA-45 left (true positives). The two images in the right column are the averaged images that recover a brain activity in regions other than BA-45 left (false positives). In this case, 81 TPs and 19 FPs are obtained.

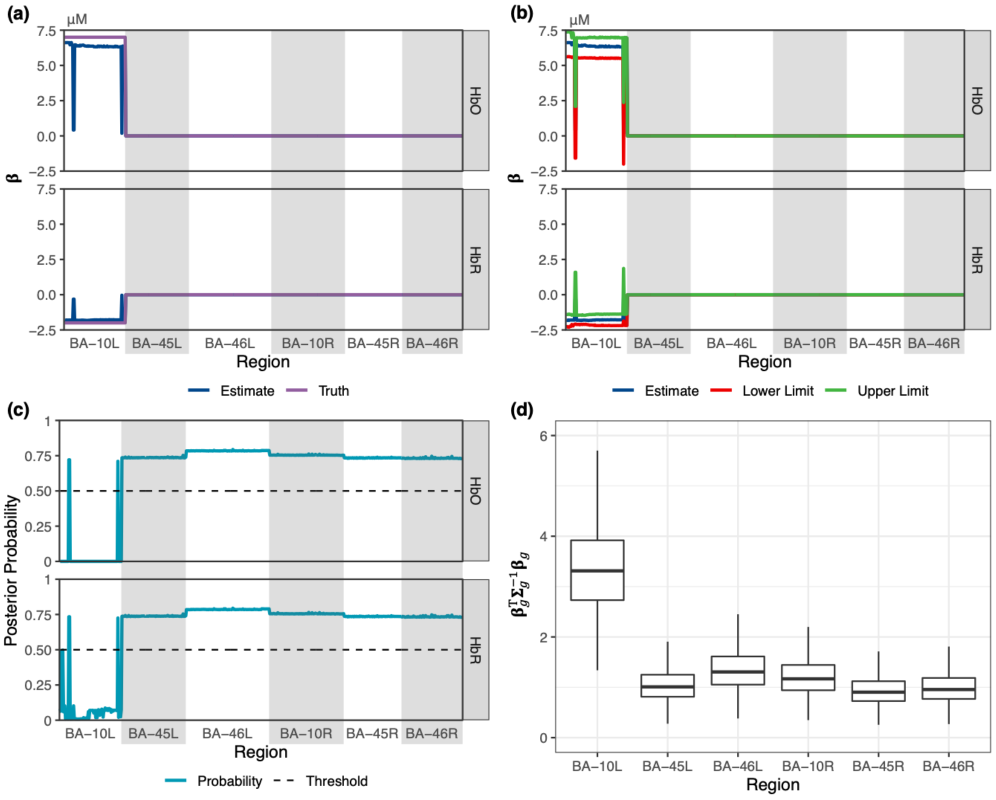


Fig. S3. Four subplots showing the statistical inference for the image reconstruction of 100 datasets with brain activity simulated in BA-10 left. (a) The line plot of the ground truth and the

estimated hemoglobin changes. (b) The estimated hemoglobin changes and the 50% CIs. (c) The posterior probability that $\beta_p$ is within the scaled neighborhood interval $\left[-\sqrt{\text{var}(\beta_p|\mathbf{X},\mathbf{y})},\sqrt{\text{var}(\beta_p|\mathbf{X},\mathbf{y})}\right]$ and the 50% probability threshold. (d) The boxplot $\boldsymbol{\beta}_g^{\mathrm{T}}\boldsymbol{\Sigma}_g^{-1}\boldsymbol{\beta}_g$ for all available Brodmann Areas. Note that each point of the lines in (a) – (c) represents the value at a voxel belonging to the region indicated on the horizontal axis and separated using the grey-shaded/white areas. Subplots (b) – (d) respectively show the statistical inference via the three approaches described in Sec. 3.2, from which we can conclude that the hemoglobin changes at most individual voxels in BA-10 left are significant based on the CI and the probability within the scaled neighborhood interval, and the brain activity in BA-10 left is significantly larger than that in the remaining ROIs as an entirety.

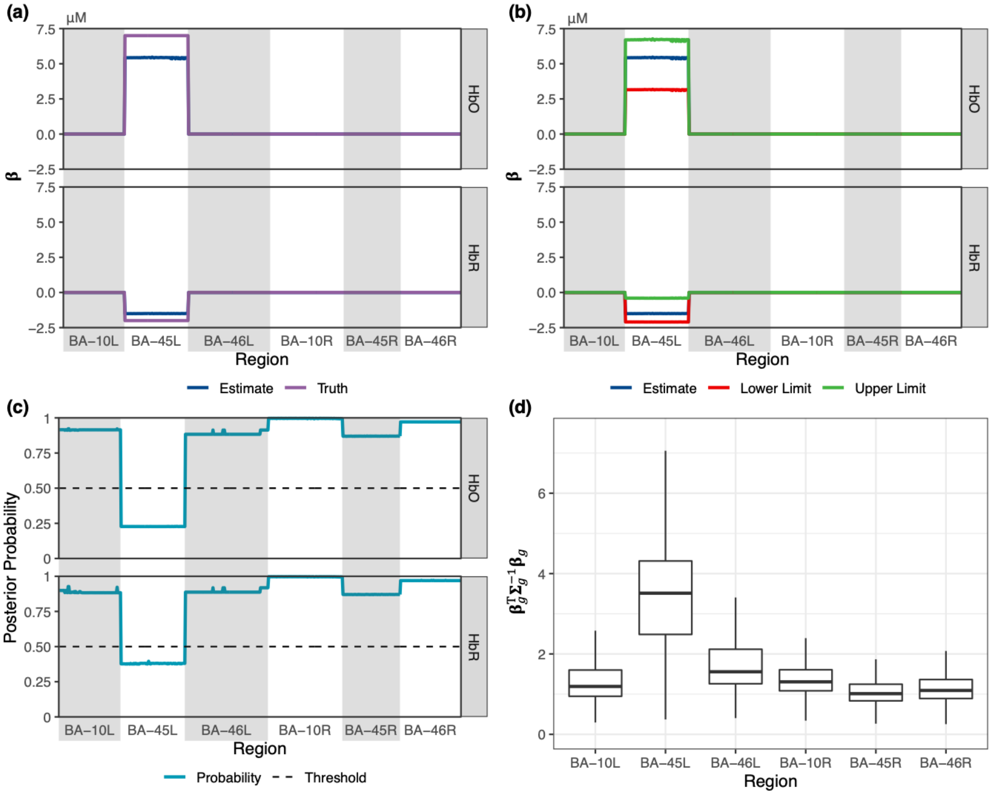


Fig. S4. Four subplots showing the statistical inference for the image reconstruction of 100 datasets with brain activity simulated in BA-45 left. (a) The line plot of the ground truth and the estimated hemoglobin changes. (b) The estimated hemoglobin changes and the 50% CIs. (c) The posterior probability that $\beta_p$ is within the scaled neighborhood interval $\left[-\sqrt{\text{var}(\beta_p|\mathbf{X},\mathbf{y})},\sqrt{\text{var}(\beta_p|\mathbf{X},\mathbf{y})}\right]$ and the 50% probability threshold. (d) The boxplot $\boldsymbol{\beta}_g^{\mathrm{T}}\boldsymbol{\Sigma}_g^{-1}\boldsymbol{\beta}_g$ for all available Brodmann Areas. Note that each point of the lines in (a) – (c) represents the value at a voxel belonging to the region indicated on the horizontal axis and separated using the grey-shaded/white areas. Subplots (b) – (d) respectively show the statistical inference via the three approaches described in Sec. 3.2, from which we can conclude that the hemoglobin changes at all individual voxels in BA-45 left are significant based on the CI and the probability within the scaled neighborhood interval, and the brain activity in BA-45 left is significantly larger than that in the remaining ROIs as an entirety.
